# Effectiveness of Obesity Management Education for Healthcare Professionals: A Rapid Review and Meta‐Analysis

**DOI:** 10.1111/cob.70113

**Published:** 2026-09-11

**Authors:** Rebecca Wu, Srividya Adapa, Maria Agaliotis, Samantha Hocking, Evan Atlantis

**Affiliations:** ^1^ School of Health Sciences Western Sydney University Campbelltown New South Wales Australia; ^2^ Boden Initiative, Charles Perkins Centre University of Sydney Sydney New South Wales Australia; ^3^ Central Clinical School, Faculty of Medicine and Health University of Sydney Sydney New South Wales Australia; ^4^ Department of Endocrinology Royal Prince Alfred Hospital Sydney New South Wales Australia

**Keywords:** clinical competence, continuing education, healthcare personnel, medical education, obesity, weight management

## Abstract

Healthcare professionals (HCPs) play a critical role in obesity management, yet many report inadequate training, low confidence and stigma‐related attitudes. While education programmes have been developed to address these issues, evidence of their effectiveness remains mixed. This study aimed to evaluate the effectiveness of structured education and training programmes in improving HCPs' practice competencies in obesity management. We searched MEDLINE, EMBASE, CINAHL, Web of Science, ERIC and Google Scholar for studies published from 2020 assessing structured obesity management education programmes for HCPs. Eligible studies reported outcomes related to practice competencies (knowledge, skills and practice behaviours, confidence, attitudes) or patient outcomes (weight loss). Standardised mean differences (SMDs) were pooled using random‐effects meta‐analysis. Heterogeneity was assessed using *I* ^2^ and *Q* statistics, and subgroup analyses explored potential sources. Studies not included in the meta‐analysis were summarised descriptively. Eighteen studies were included from 1683 records. Pooled analysis of four RCTs and three non‐RCTs showed a significant improvement in practice competencies (SMD = 0.41, 95% CI 0.12–0.70, *p* < 0.01) with high heterogeneity (*I* ^2^ = 60%). Effects were significant in RCTs (SMD = 0.53, 95% CI 0.10–0.95, *p* = 0.01), but not non‐RCTs (SMD = 0.25, 95% CI −0.04 to 0.55, *p* = 0.09). Interventions lasting ≥ 4 h produced larger, significant effects (SMD = 0.68, 95% CI 0.30–1.07, *p* < 0.01), while shorter interventions did not. Quasi‐experimental studies supported improvements across HCP competencies and occasionally, patient outcomes. Structured education and training programmes may improve HCP competencies in obesity management, particularly confidence, with stronger effects generally observed in longer, more comprehensive interventions (≥ 4 h).

## 1 Introduction

Obesity is a complex chronic disease characterised by excess adiposity that significantly impairs bodily function and increases the risk of adverse health outcomes [1]. In 2021, an estimated 2.1 billion adults globally were living with overweight or obesity, with prevalence projected to reach 3.8 billion adults by 2050 [2]. The rising prevalence highlights the need for effective public health strategies, especially given obesity's association with a wide range of medical, mental and functional complications [1, 3, 4, 5]. As a result, the growing health and economic consequences of obesity necessitate effective intervention from skilled healthcare professionals (HCPs) as a central component of mitigation efforts [5, 6, 7].

The effectiveness of such interventions is largely dependent on the preparedness of HCPs. Although HCPs play a central role in the prevention and management of obesity [8, 9], research consistently highlights gaps in their readiness to deliver obesity care [10, 11, 12, 13]. Studies frequently report that HCPs experience difficulties with low confidence and self‐efficacy, demonstrate knowledge gaps and may hold stigmatising attitudes towards individuals living with obesity [13, 14, 15]. To address these challenges, it is critical to implement comprehensive education and ongoing professional development to ensure HCPs are well‐equipped to deliver evidence‐based care.

While the need for educational interventions is clear, their reported effectiveness varies considerably. Educational interventions in published literature vary widely in formats and intensity, from short educational videos [16, 17] and online modules [18, 19, 20], to more extensive multi‐day, in‐person programmes [21, 22]. Findings across studies are inconsistent, with mixed results reported for key outcomes such as HCP knowledge [17, 23], skills [23, 24, 25], confidence [16, 19, 21] and attitudes [17, 21, 26]. Furthermore, the impact on patient outcomes is seldom assessed.

This inconsistency highlights a critical gap in the existing literature. While the value of ongoing professional development in obesity care is widely acknowledged [12, 27, 28], a key evidence gap persists. There is no recent systematic review or meta‐analysis that comprehensively evaluates the effectiveness of structured obesity management training programmes for practising HCPs. Previous reviews have primarily focused on pre‐licensure students or medical practitioners, often describing intervention characteristics without evaluating their impact [27, 28]. For example, a recent scoping review reported improvements in skills, self‐efficacy and attitudes, but was limited to current and prospective medical doctors in Canada [27]. Similarly, a recent systematic review also found predominantly positive outcomes, though only counselling confidence was supported by high‐quality, consistent evidence and the review focused exclusively on physicians, residents and medical students [28]. Since then, additional studies have emerged, including those targeting other types of HCPs [17, 18, 25, 26, 29, 30, 31]. Therefore, a comprehensive and up‐to‐date evaluation of educational programmes is essential to inform the development of training that meets the current demands of obesity management, including the appropriate use of new therapeutic agents [32, 33].

To address this knowledge gap, we conducted a rapid review with meta‐analysis to synthesise and evaluate recent evidence on the effectiveness of structured education and training programmes in obesity management for HCPs. Specifically, this review examined studies published in the past 5 years, assessing their impact on HCPs' knowledge, skills, confidence, attitudes towards individuals living with obesity, and, where reported, patient outcomes.

## 2 Materials and Methods

This rapid systematic review methodology was developed following established methodological frameworks, including guidance from the Centre for Review and Dissemination's (CRD) Guidance for Undertaking reviews in Healthcare [34], the Joanna Briggs Institute (JBI) methodology [35] and reported in accordance with the Preferred Reporting Items for Systematic reviews and Meta‐Analyses (PRISMA) statement [36].

### 2.1 Eligibility Criteria

We developed the research question and study eligibility criteria using a modified version of the Population, Interventions, Comparators, Outcomes (PICO) framework [37] (Table 1). Studies were included if they involved practising HCPs (such as doctors, nurses and allied health professionals) in any healthcare setting. Studies involving students were excluded. Eligible interventions were structured education and training programmes specifically focused on obesity management. For the purposes of this review, structured education and training programmes were defined as interventions with a defined set of learning objectives, a planned curriculum or content structure specific to obesity management, and a specified format, duration and mode of delivery. This definition was applied irrespective of whether a formal, nationally recognised obesity education competency framework existed, recognising that such frameworks vary across the countries represented in this review. Interventions lacking a defined educational structure, such as ad hoc clinical mentoring, informal peer discussion or attendance at general continuing professional development (CPD) activities without a specific obesity‐focused curriculum, were excluded. Formal outcome measurement was treated as a separate inclusion criterion related to study evaluation rather than intervention design and was therefore assessed independently of the structured education definition. Interventions that addressed general health or lifestyle education without a specific focus on obesity management were excluded.

**TABLE 1:** Inclusion criteria using the PICO framework.

Population (P)	Health care professionals (for example, doctors, nurses, allied health professionals) in any setting. Does not include students
Intervention (I)/Exposure	Structured education and training programmes on obesity management
Comparator/Control (C)	Not applicable; studies with or without a control group are included
Outcomes (O)	Practice competency outcomes: improvement in healthcare professionals' knowledge, skills and confidence in managing obesity, including available treatments and interventions (e.g., behavioural, pharmacotherapy, bariatric surgery, obesity stigma) AND/OR Patient outcomes: weight loss, adherence to treatment, obesity stigma and management of comorbidities Eligible studies formally measured outcomes pre‐ and post‐intervention (with or without a comparator)
Time (T)	Studies published in the last 5 years (from 2020)

We included studies with and without a control group. Recognising the practical challenges of conducting randomised controlled trials (RCTs) for education interventions, this approach allowed us to capture a broad range of available evidence regarding obesity education for HCPs. Eligible outcomes included changes in HCP practice competencies, specifically knowledge, skills, confidence and attitudes related to obesity management. Studies reporting patient outcomes, such as weight loss, treatment adherence and the improved management of comorbidities, were also included. Eligibility was further restricted to studies that formally evaluated the impact of the intervention using pre‐ and post‐intervention measurement, with or without a control or comparison group. This outcome‐evaluation requirement was applied independently of the criteria used to define a structured education and training programme. Studies describing structured obesity education programmes without any formal assessment of HCP‐ or patient‐based outcomes were excluded, as they could not contribute to an evaluation of intervention effectiveness. To ensure the relevance of the evidence, we limited our search to studies published within the last 5 years (from 2020 onwards) (Table 1).

### 2.2 Information Sources, Search Strategy and Study Selection

The search strategy was developed in consultation with subject experts (E.A. and M.A.) and an academic librarian. We searched the titles and abstracts of papers using keywords derived from our PICO criteria in the following online databases: MEDLINE via Ovid or via PubMed, Excerpta Medica Database (EMBASE) EBSCOhost, Cumulative Index to Nursing & Allied Health Literature (CINAHL), Web of Science and ERIC EBSCOhost or ProQuest, as well as Google Scholar (a specialised search engine). The search covered articles published between January 2020 and March 2025. The full search strategy and results are provided in Supporting Information S1.

All identified records were exported to EndNote 20, and duplicate records were removed where possible. The titles and abstracts of the identified records were first screened by one reviewer (R.W.), and those that did not meet the inclusion criteria were removed. The full texts of the remaining studies were then assessed for eligibility against the inclusion criteria by the same reviewer (R.W.), and ineligible studies were excluded. Any uncertainties regarding study selection were resolved through discussion with the other authors (E.A. and M.A.).

### 2.3 Data Extraction and Risk of Bias Assessment

A standardised data extraction form was jointly developed by the authors (R.W., E.A. and M.A.) to determine which variables to extract. Two reviewers (R.W. and E.A.) independently extracted data from the included studies into a spreadsheet in Microsoft Word, and a third author (S.A.) verified the extracted data. Any discrepancies were resolved through discussion between the authors.

We extracted data on study characteristics (author, year of publication, study design and country); population and setting (sample size, inclusion and exclusion criteria, population); intervention (format and mode of delivery, topics covered, duration and frequency, and type of control); outcomes measured; data analysis and results and author's conclusions. For outcomes with more than five individual measurements (e.g., multi‐item questionnaires that lacked a global score), we limited extraction to the five components most relevant to the intervention's primary aims to avoid over‐representation of single studies. For missing or unclear data, study authors were contacted by email up to twice over a 4‐week period before proceeding with the available information.

A risk of bias assessment was conducted on the included studies by two reviewers (R.W. and S.A.) using the JBI critical appraisal tool for RCTs [38] and quasi‐experimental studies [39], including non‐RCTs and before‐and‐after studies. Any discrepancies were resolved through discussion between the authors (R.W., E.A. and S.A.).

### 2.4 Data Synthesis

Study characteristics, including study design, country, sample size, population, intervention delivery and content, and outcomes measured, were narratively summarised. To evaluate the effectiveness of interventions on HCP practice competencies, a meta‐analysis including both RCTs and non‐RCTs was conducted. This inclusive approach allowed for a more comprehensive synthesis, given the limited availability of robust RCT data in this area and the relevance of non‐RCTs for evaluating interventions in real‐world professional development settings.

All statistical analyses were conducted in R (version 4.5.1) using the meta package (6.0‐0). Primary outcomes were analysed using random‐effects meta‐analyses to account for potential heterogeneity between studies. Effect sizes for continuous outcomes were calculated as SMD, expressed as Cohen's *d*. For outcomes reported as ratios or frequencies [24], the Phi coefficient was used [40]. Heterogeneity was quantified using Cochran's *Q* test and the *I* ^2^ statistic, which describes the percentage of total variation across studies attributable to true heterogeneity rather than chance. Forest plots were generated to visually represent the results of the meta‐analyses. Each plot displays the individual study effect sizes with 95% confidence intervals and corresponding study weights. The overall pooled effect size and its 95% confidence interval are presented as a diamond at the bottom of the plot.

The primary outcome was practice competencies, operationally defined as improvements in HCP outcomes (knowledge, skills, confidence, attitudes, practice behaviours) and patient outcomes (e.g., weight loss). For meta‐analysis, one outcome was selected from each study, prioritising domains most frequently assessed across studies. To ensure consistency in interpretation, effect sizes for attitude‐related outcomes were multiplied by −1 where necessary, so that positive effect sizes consistently indicated improvement (e.g., a reduction in bias). This standardisation procedure follows the Cochrane Handbook's guidance to ensure all effects are ‘pointing’ in the same direction before pooling [41].

Subgroup analyses were conducted based on predefined study characteristics, including study design (RCT vs. non‐RCT) and intervention duration. A 4‐h threshold was selected to categorise intervention duration, as this value provided the most even distribution of studies for meaningful comparison. Additional subgroup analyses were performed by outcome domain (skills and behaviours, attitudes, knowledge and confidence), including only studies that reported outcomes within the relevant domain and selecting one representative outcome per study. Sensitivity analyses were also performed by omitting one study at a time from the overall pooled analysis to assess robustness of the findings. Studies not included in the meta‐analysis (quasi‐experimental studies) were descriptively synthesised.

## 3 Results

### 3.1 Study Selection

**FIGURE 1:**
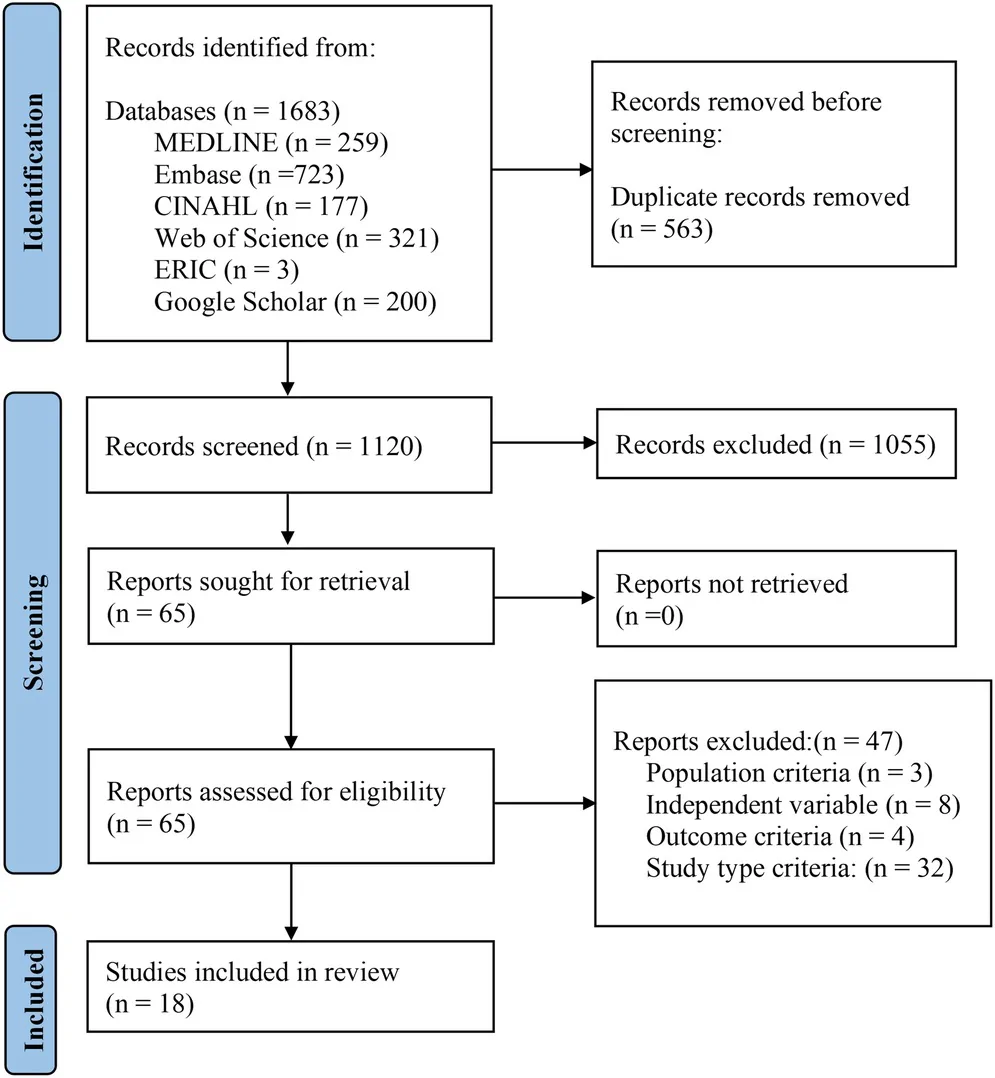
PRISMA flow diagram of the study selection process.

Our study selection process identified 18 eligible studies from an initial 1683 records. After removing 563 duplicates, 1120 records were screened by title and abstract, of which 1055 were excluded. The remaining 65 articles underwent full‐text screening, resulting in a further 47 exclusions. A flow diagram detailing the study selection process and reasons for exclusion is presented in Figure 1.

### 3.2 Study Characteristics

A detailed summary of the characteristics of the included studies is presented in Supporting Information S2. In total, 18 studies published between 2020 and 2025 were included, which described and evaluated obesity management training programmes for HCPs. Of these, four were RCTs [17, 18, 23, 26], one was a non‐RCT [19], 10 used a before‐and‐after design [16, 20, 21, 22, 29, 30, 31, 42, 43, 44] and three had both a non‐RCT and a before‐and‐after study design [24, 25, 44]. The studies were conducted across various regions, with nine from the United States of America (USA) [17, 19, 20, 22, 24, 30, 42, 43, 45], five from Canada [16, 21, 25, 29, 44] and one each from Australia [18], Indonesia [23], Israel [26] and Malaysia [31].

The included studies had sample sizes ranging from seven to 506 participants. Two studies had fewer than 10 participants [20, 29], six studies had 10–50 participants [19, 21, 22, 25, 30, 31], seven studies had 50–100 participants [16, 17, 18, 23, 42, 43, 44] and three studies included more than 100 participants [24, 26, 45]. Three studies included all types of health care professionals [16, 20, 45], while the remaining 15 studies focused on specific HCP groups, such as physicians, nurses, allied health professionals (e.g., dietitians, physical therapists, social workers, occupational therapists and pharmacists), medical assistants and medical centre employees [17, 18, 19, 21, 22, 23, 24, 25, 26, 29, 30, 31, 42, 43, 44].

Educational interventions were delivered via multiple modes in six studies [19, 21, 22, 23, 39, 40], and a singular mode in 12 studies [16, 17, 18, 20, 24, 25, 26, 29, 30, 31, 42, 45]. Delivery methods included self‐paced learning activities and modules [18, 19, 20, 23, 26, 30, 43, 44], workshops and seminars [19, 21, 22, 24, 25, 29, 31, 42, 43, 45], educational videos [16, 17] and a discussion forum [21]. The interventions were composed of features including videos and lectures [16, 17, 18, 19, 20, 21, 23, 30, 31, 44, 45], case studies/vignettes [18, 19, 21, 22, 23, 25, 29, 30, 31, 42, 43, 45], quizzes and assignments [18, 19, 23, 26, 30], discussions with other participants [17, 19, 21, 22, 23, 25, 31, 44], patient feedback from consultations [18], patient visits [21, 22] and patient panel discussions [45]. Six interventions were delivered online [18, 20, 23, 26, 30, 44], seven were in‐person [17, 22, 24, 25, 31, 42, 45], three had both online and in‐person components [19, 21, 43], one was accessible both online and in‐person [29] and one study did not specify the delivery mode [16].

The duration of the self‐paced learning activities and modules varied widely, ranging from a brief 15‐min activity [26, 43] to modules requiring 2–10 h to complete [18, 19, 20, 23, 30]. For longer modules, participants were given between 4 weeks to 3 months to finish the content. Workshops and seminars also varied in length, from short sessions of 30 min to 2 h [19, 21, 24, 29, 42, 43], to half‐day [23, 25, 45], full day [31] and multi‐day events [21, 22]. In four studies, workshops/seminars were delivered on a recurring basis: one study held weekly online meetings over a month [23], while others conducted sessions monthly [19, 21] or every 5 weeks [22], over the course of 3–12 months. Two studies used educational videos as the primary intervention, with durations of 6 min [16] and 17 min [17]. One intervention included a discussion forum that remained active for 1 year [21]. One study did not specify the duration or frequency of its intervention [44].

The interventions covered a range of topics, including general knowledge of obesity; lifestyle, pharmacological and surgical interventions for weight management; weight stigma and bias and practical counselling strategies. General knowledge content encompassed an overview of overweight and obesity, the biological complexity of obesity, risk factors and psychological aspects of weight management [17, 18, 20, 22, 24, 25, 26, 30, 31, 43, 44, 45]. Lifestyle interventions focused on dietary approaches, physical activity and behavioural modifications [17, 18, 19, 21, 22, 23, 25, 26, 31, 42, 43, 44]. Pharmacological and surgical content addressed the use of medication and bariatric surgery [17, 18, 19, 21, 22, 25, 26, 30, 31, 44, 45]. Content on weight stigma and bias addressed personal beliefs on weight, prevalence and impact of weight bias, strategies to reduce stigma and patient experiences with stigma [17, 18, 19, 20, 24, 25, 26, 30, 43, 45]. Counselling strategies encompassed behaviour change techniques, communication approaches when addressing weight, patient assessment, nutrition counselling, care plan development, goal setting and telehealth [16, 17, 18, 21, 22, 23, 24, 25, 29, 30, 31, 42, 44]. Some interventions also included content that was specific to certain types of health professionals, including physiotherapists, occupational therapists and community pharmacists [18, 30, 31]. Four interventions included content relevant for the management of overweight and obesity in children and adolescents [16, 21, 23, 29].

The outcomes measured across studies varied. Knowledge of obesity management was assessed in six studies [17, 22, 23, 25, 31, 42], practice behaviours (e.g., counselling skills and patient referrals) in nine studies [19, 21, 23, 24, 25, 29, 42, 44, 45], and confidence or perceived self‐efficacy in weight management in seven studies [16, 18, 19, 21, 29, 30, 43]. Attitudes towards obesity and weight bias were evaluated in 11 studies [17, 18, 20, 21, 22, 24, 26, 30, 31, 43, 45], while two reported patient outcomes such as weight loss and waist circumference [21, 22]. Ten studies included follow‐up assessments to examine the sustained effects of the intervention, with follow‐up periods ranging from 1 month to one year [16, 17, 19, 21, 24, 26, 29, 31, 42, 45].

### 3.3 Risk of Bias Within Studies

An assessment of the risk of bias within each study indicated differing levels of methodological quality, with full details available in Supporting Information S3 and S4. The four RCTs clearly met between 58% and 89% of the quality criteria [17, 18, 23, 26]. Of the 14 non‐RCTs and/or before‐and‐after studies, two studies met 78% or more of the quality criteria [24, 45], nine studies met 71% of the quality criteria [16, 20, 22, 29, 30, 31, 42, 43, 44] and three studies met 67% or less of the quality criteria [19, 21, 25].

### 3.4 Synthesis With Meta‐Analysis

Eight studies with a control or comparison group were identified as potentially eligible for meta‐analysis [17, 18, 19, 23, 24, 25, 26, 45]. Of these, one study [45] was excluded due to insufficient summary data to calculate the SMD and pooled standard deviation required for meta‐analysis.

#### 3.4.1 Overall Effect Size on Practice Competencies

**FIGURE 2:**
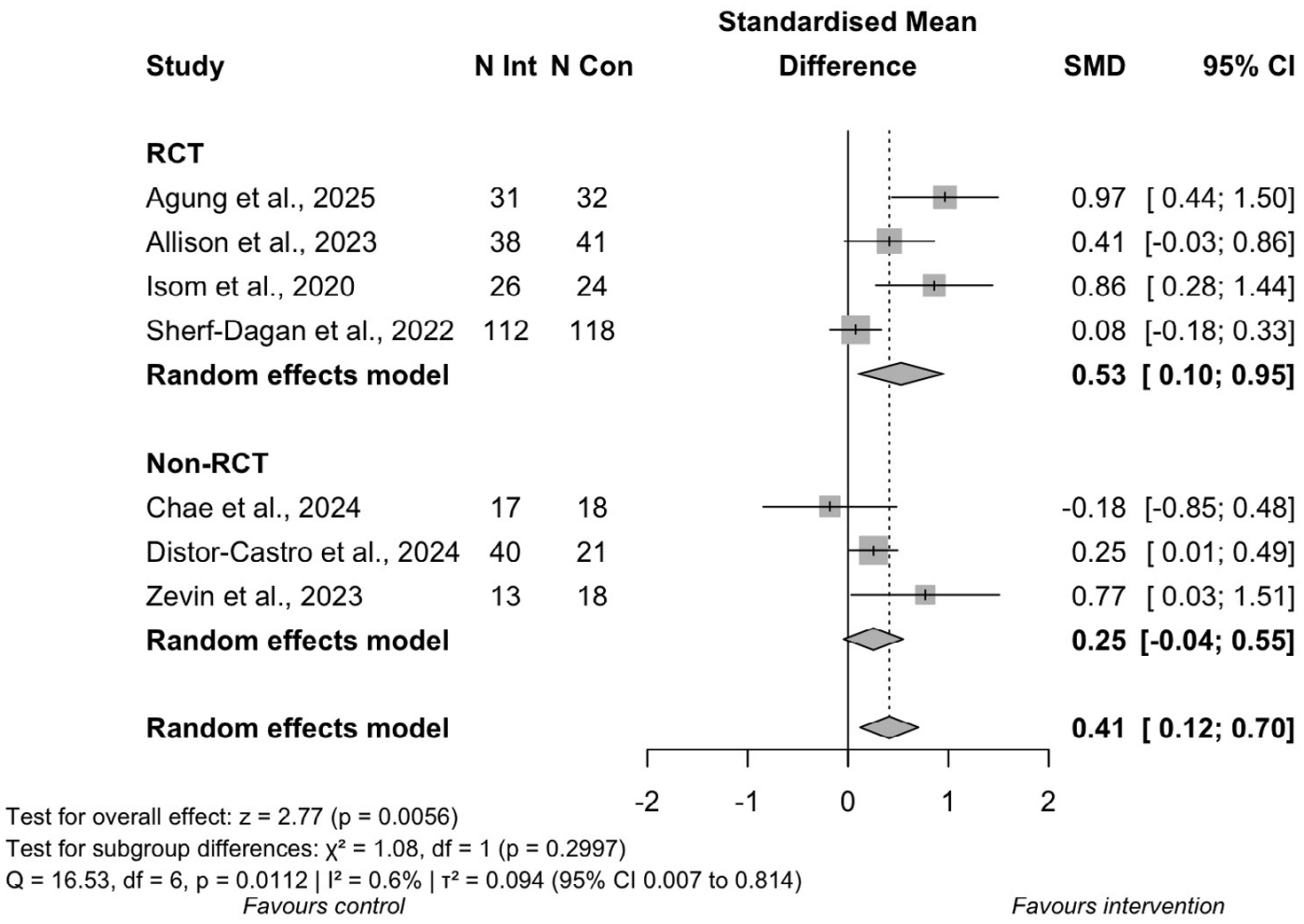
Forest plot of pooled standardised mean difference with subgroup analysis by study design.

The pooled analysis included four RCTs [17, 18, 23, 26] and three non‐RCTs [19, 24, 25] (Figure 2). Meta‐analysis yielded a statistically significant moderate positive effect of the interventions on practice competencies (SMD = 0.41, 95% CI 0.12–0.71, *p* < 0.01). Substantial heterogeneity was detected (*I* ^2^ = 60%, *Q* = 16.67, *p* = 0.01), suggesting considerable variability in standardised mean differences (SMDs) across studies, though consistently favouring the intervention.

#### 3.4.2 Subgroup Analysis

When stratified by study design, RCTs yielded a moderate and statistically significant positive effect (SMD = 0.53, 95% CI 0.10–0.95, *p* = 0.01), whereas non‐RCTs showed a smaller, non‐significant effect (SMD = 0.25, 95% CI −0.04 to 0.55, *p* = 0.09, Figure 2).

**FIGURE 3:**
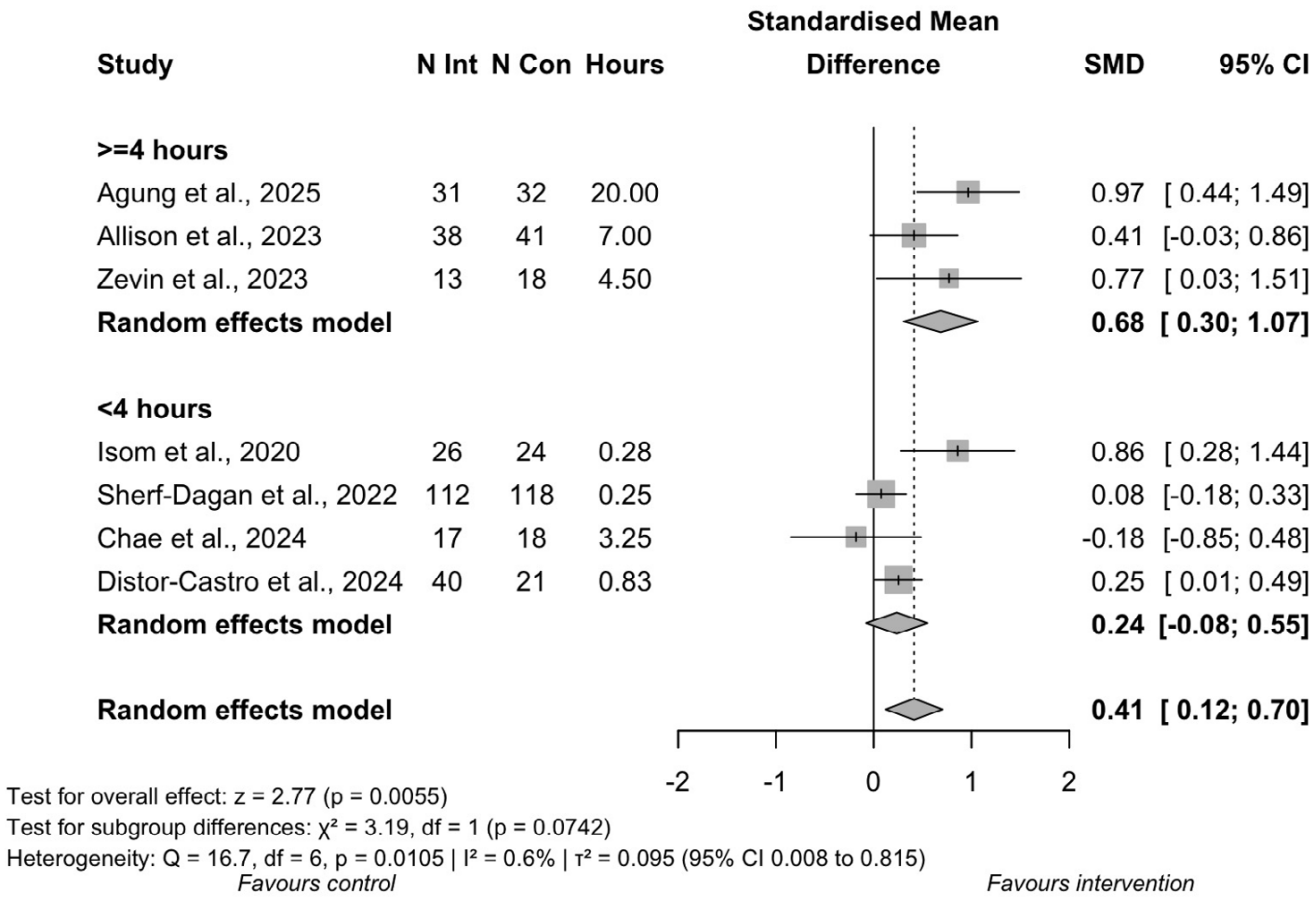
Forest plot of pooled standardised mean difference with subgroup analysis by intervention duration.

Analysis by training duration indicated that interventions lasting ≥ 4 h had a moderate and statistically significant positive effect (SMD = 0.68, 95% CI 0.30–1.07, *p* < 0.01), while those < 4 h showed a smaller and non‐significant SMD (0.24, 95% CI −0.08 to 0.55, *p* = 0.14, Figure 3). These findings suggest that the overall positive SMD was primarily driven by longer interventions.

Analysis by outcome domain yielded a statistically significant improvement only in confidence. Pooled analysis of the two studies assessing confidence [18, 19] showed a large and significant SMD (1.79, 95% CI 0.07–3.50, *p* = 0.04). In contrast, pooled analyses for other domains yielded moderate but non‐significant SMDs: skills and behaviours (four studies: SMD = 0.44, 95% CI −0.03 to 0.92, *p* = 0.07), attitudes (three studies: SMD = 0.39, 95% CI −0.05 to 0.82, *p* = 0.08) and knowledge (two studies: SMD = 0.54, 95% CI −0.51 to 1.58, *p* = 0.31). Forest plots for these subgroup analyses are provided in Supporting Information S5.

#### 3.4.3 Sensitivity Analysis

Sensitivity analyses, conducted by omitting one study at a time from overall meta‐analysis, confirmed the robustness of the findings. The exclusion of any single study did not meaningfully alter the overall SMD or its statistical significance. Effect sizes ranged from 0.31 to 0.49 and remained statistically significant across all iterations. Forest plots for these analyses are presented in Supporting Information S6.

### 3.5 Synthesis Without Meta‐Analysis

A total of 13 studies with a before‐and‐after study design investigated the effectiveness of educational interventions by comparing outcomes prior to and following the intervention [16, 20, 21, 22, 24, 25, 29, 30, 31, 42, 43, 44, 45].

#### 3.5.1 Knowledge

Two studies reported positive within‐group changes in self‐rated knowledge. A US‐based study found a significant 30%–70% improvement in self‐rated knowledge across multiple obesity‐related domains (e.g., co‐morbidities, pharmacotherapy, nutrition, bariatric surgery) [22]. A Canadian study reported that 15 of 16 participants felt the intervention enhanced their knowledge, although no pre‐intervention measurements were taken [25].

Findings were more variable when knowledge was assessed objectively. A Malaysian study reported a significant improvement of approximately 18% in obesity‐related knowledge [31], while a US study found a significant 27% post‐intervention increase in nutrition counselling knowledge, which declined at 2‐month follow‐up [42].

#### 3.5.2 Practice Behaviours and Skills

Six studies assessed within‐group changes in HCPs' practice behaviours and skills, with mixed results. A Canadian study observed significant increases at 1‐year follow‐up in behaviours such as using readiness assessments, performing weight and waist circumference measurements, and using tools like food diaries and pedometers, with some behaviours increasing more than fourfold [21]. A US‐based study reported a significant 20% improvement in self‐reported competence across multiple obesity management domains, including weight discussions, prescribing medication and dietary and physical activity recommendations [22]. Another Canadian study found a 3%–13% improvement in self‐reported clinical skills, though no statistical significance was calculated for these results [29]. A US‐based study reported a 30%–60% increase in nutritional counselling behaviours at 2‐month follow‐up, but found no significant changes in documentation of obesity‐related care in electronic health records [42]. A third Canadian study found high intent in HCPs to change their practice; however, there was no significant change in measured referral rates for medical and/or surgical weight loss [25]. Lastly, a fourth Canadian study reported changes in treatment approaches, including a reduction in lifestyle interventions (by 30%) and increased use of pharmacotherapy (by 83%) and bariatric surgery (by 33%), without statistical reporting [45].

#### 3.5.3 Confidence

Five studies reported positive within‐group changes in HCP's confidence. A Canadian study found a significant 40%–60% improvement in confidence in managing obesity and providing lifestyle and pharmacotherapy advice [21]. Another Canadian study reported a significant 15% increase in self‐efficacy in initiating weight‐related conversations, which was sustained at 6 months [16]. A third Canadian study observed a 20%–65% gain in self‐efficacy in obesity‐related communication, although statistical significance was not reported [29]. A US‐based study reported a significant 10% increase in comfort working with patients with obesity [43]. Lastly, another US study reported a significant 35%–55% improvement in self‐rated competence in evaluating and treating patients with obesity, understanding psychosocial comorbidities and using bariatric equipment [30].

#### 3.5.4 Attitudes

Eight studies assessed changes in HCP's attitudes towards obesity, weight‐bias and stigma, with mixed results. Three US studies and one Malaysian study reported significant improvements (10%–55%) in awareness of weight‐bias and stigma, as well as positive beliefs about obesity management [20, 30, 31, 43]. Another US study showed improved attitudes towards patients, though the effects varied by profession [21]. Three studies reported limited or no change. Two US studies found that attitudes and weight‐bias remained stable across the follow‐up period [22, 45], while another US study reported a 7%–9% gain in empathy and critical perspectives but no change in beliefs about the causes of obesity [24].

#### 3.5.5 Patient Outcomes

Two studies assessed within‐group changes to patient outcomes, both reporting positive results. A Canadian study observed a statistically significant mean weight loss of 4.5 kg (*p* < 0.0001) and a 4.3 cm reduction in waist circumference (*p* < 0.0001) after 1 year [21]. Similarly, a US study reported a statistically significant mean weight loss of 4.5 kg (95% CI 3.0–6.0) over a 12‐month intervention [22].

## 4 Discussion

This rapid review and meta‐analysis found that structured education and training programmes are effective in improving HCPs' competencies in obesity management, consistent with findings from other recent reviews and studies [27, 28, 46]. Our review directly addresses the evidence gap identified in the introduction by providing a current synthesis of evidence on the effectiveness of obesity management education and training programmes specifically for practising HCPs. Overall, the interventions demonstrated positive effects on competencies, with stronger outcomes observed in longer and more comprehensive programmes. This suggests that interventions of longer duration (e.g., ≥ 4 h) are required to develop the complex knowledge and skills necessary for effective obesity care. The more robust effect seen in longer interventions may potentially reflect their capacity to address not only the clinical aspects of the obesity, but also equally important elements such as patient communication, motivational interviewing and strategies to reduce weight bias. To our knowledge, this is a novel finding, as recent studies have not examined the relationship between intervention duration and effectiveness [46, 47, 48], apart from an older review which reported that repetitive interventions produced better outcomes than one‐off interventions [49].

The positive effect of obesity management training programmes on competencies has important implications for clinical practice. The evidence suggests that a key benefit of structured education and training programmes is its ability to enhance HCPs' self‐efficacy in managing obesity, which is a significant barrier to the provision of effective care [50]. In our review, confidence outcomes showed the largest improvement following educational interventions, aligning with broader literature demonstrating that learning experiences often strengthen confidence [51, 52, 53]. Greater HCP confidence may lead to more frequent and open discussions with patients about weight, potentially increasing the initiation of evidence‐based treatments. Findings from the quasi‐experimental studies reinforced these results, demonstrating positive changes in practice behaviours, knowledge and attitudes, even in the absence of a control group. For instance, studies reported increased frequency of patient weight and waist circumference measurements following training [21], suggesting that educational programmes can translate knowledge gains into practice changes. However, it is important to note the mixed results across other competencies within the meta‐analysis, with non‐significant effects found for skills, attitudes and knowledge. This may be due to the limited number of studies within these subgroups or methodological differences in how these outcomes were measured.

It is also worth noting that ‘structured’ education, as defined by curriculum and format, does not guarantee equivalent rigour, content or depth across settings. Formal obesity competency frameworks have been developed in several jurisdictions represented in this review, including the Canadian Obesity Education Competencies, which are based on the CanMEDS framework; the US‐based Obesity Medicine Education Collaborative (OMEC) competencies and Australia's National Framework for Clinical Obesity Services (NACOS) [54, 55, 56]. The NACOS framework adapts OMEC standards of care and competencies to the Australian context and explicitly recommends routine education and training in specialist obesity management for medical students and HCPs [57]. Variability in the availability, scope, adoption and implementation of these frameworks across countries and healthcare systems may have contributed to the heterogeneity observed among the studies included in this review. Future programme development and evaluation may benefit from aligning structured education content with existing national competency frameworks, where available, to improve consistency and comparability across studies and settings.

Our review has several strengths and limitations. Methodologically, the inclusion of both RCTs and quasi‐experimental designs allowed for a complete understanding of the evidence base, while the application of meta‐analysis provided a quantitative synthesis of the pooled effects. By focusing on studies published in the last 5 years, this review provides a timely snapshot of the most recent evidence. The comprehensive search across multiple disciplinary databases, supplemented by Google Scholar, ensured that a wide range of relevant literature was captured.

Conversely, while time‐efficient, the rapid review methodology relied on a single reviewer for initial screening, which may have introduced bias. Substantial heterogeneity across the included studies complicated interpretation of the pooled results and underscores the need for more standardised interventions and outcome measures. Furthermore, many studies relied on self‐reported outcomes, and few included long‐term (e.g., > 1 year) follow‐up or patient‐level outcomes. Consequently, there is insufficient evidence to determine whether improvements in HCP competencies translate into better patient health outcomes, such as weight loss or improved comorbidity management.

## 5 Conclusion

Structured education and training programmes show promise for improving HCP competencies in obesity management, with the strongest and most consistent evidence observed for improvements in confidence. Interventions of 4 h or longer generally demonstrated larger effects than shorter interventions, suggesting a potential benchmark for future programme design. However, evidence for improvements in skills, attitudes and knowledge remains inconsistent, and it is not yet clear whether HCP education translates into improved patient outcomes. Future studies should employ standardised methodologies, larger samples and longer follow‐up periods to better clarify these effects and their sustainability over time.

## Author Contributions

R.W. was responsible for designing the review protocol, writing the protocol and report, conducting the search, screening potentially eligible studies, extracting and analysing data, conducting the risk of bias assessment, interpreting results and updating reference lists. S.A. was responsible for extracting data, conducting the risk of bias assessment and reviewing the report. M.A. was responsible for designing the review protocol, reviewing the report and overall project supervision. S.H. was responsible for designing the review protocol, reviewing the report and overall project supervision. E.A. was responsible for designing the review protocol, reviewing the protocol and report, extracting and analysing data, reviewing the report and overall project supervision.

## Ethics Statement

This study was granted ethics exemption by the Western Sydney University Human Research Ethics Committee (Ethics Reference: EX‐H16601).

## Conflicts of Interest

R.W. is supported by the Western Sydney University Postgraduate Research Scholarship, jointly funded by Western Sydney University and the National Association of Clinical Obesity Services (NACOS) and receives payment for providing Secretariat services to NACOS. S.A. is supported by Western Sydney University Postgraduate Research Scholarship. S.H. holds an honorary position as President of NACOS. E.A. holds an honorary position as Secretary of NACOS.

## Supporting information


**Supporting Information: S1.** Search strategy.
**Supporting Information: S2** Characteristics and summary of included studies.
**Supporting Information: S3** Risk of bias assessment of RCTs.
**Supporting Information: S4** Risk of bias assessment of quasi‐experimental studies.
**Supporting Information: S5** Subgroup analyses by outcome domain.
**Figure S1:** Forest plot of pooled effect sizes for the knowledge domain subgroup.
**Figure S2:** Forest plot of pooled effect sizes for the skills and behaviours domain subgroup.
**Figure S3:** Forest plot of pooled effect sizes for the confidence domain subgroup.
**Figure S4:** Forest plot of pooled effect sizes for the attitudes domain subgroup.
**Supporting Information: S6** Sensitivity analyses of the primary meta‐analysis.
**Figure S1:** Forest plots from sensitivity analysis, showing pooled effect sizes with each study omitted in turn.

## Data Availability

The data that support the findings of this study are available from the corresponding author upon reasonable request.
