# Supplementary material for: Effectiveness of Obesity Management Education for Healthcare Professionals: A Rapid Review and Meta‐Analysis
Source: Clin Obes. 2026 Sep 11;16(5):e70113. doi: 10.1111/cob.70113 (PMC13569472; doi:10.1111/cob.70113)
Supplement: Supplementary file 1 [file COB-16-e70113-s001.pdf]

Effectiveness of Obesity Management Education for Healthcare Professionals: A Rapid Review and Meta-Analysis

Rebecca Wu[1], Srividya Adapa[1], Maria Agaliotis[1], Samantha Hocking[2,3,4], Evan Atlantis[1]

[1]School of Health Sciences, Western Sydney University, Campbelltown, NSW, Australia

[2]Boden Initiative, Charles Perkins Centre, University of Sydney, NSW, Australia

[3]Central Clinical School, Faculty of Medicine and Health, University of Sydney, NSW, Australia

[4]Department of Endocrinology, Royal Prince Alfred Hospital, NSW, Australia

Corresponding author:

Name: Rebecca Wu

Address: Western Sydney University, Campbelltown, NSW 2560, Australia

Telephone number: +61 2 9174 6928

Email: 22202014@student.westernsydney.edu.au, rebecca9wu@gmail.com

Supplementary Materials

Table of contents

Supplementary Material S1: Search strategy 3
Ovid MEDLINE(R) ALL <1946 to present> 3
Embase via OvidSP <1947 to present> 4
Web of Science 5
ERIC 6
CINAHL 7
Google Scholar 8
List of excluded studies with reasons 8
Supplementary Material S2: Characteristics and summary of included studies 10
Supplementary material S3: Risk of Bias assessment of RCTs 36
Supplementary material S4: Risk of Bias assessment of quasi-experimental studies 37
Supplementary material S5: Subgroup analyses by outcome domain 39
Supplementary material S6: Sensitivity analyses of the primary meta-analysis 43

Supplementary Material S1: Search strategy

Ovid MEDLINE(R) ALL <1946 to present>

#	Query	Results from 17th March 2025
1	health personnel/ or allied health personnel/ or medical staff/ or nurses/ or nursing staff/ or nutritionists/ or occupational therapists/ or pharmacists/ or physical therapists/ or physicians/	283,372
2	(((Health* or medic*) adj3 (professional* or staff* or worker* or provider* or trainee* or practitioner* or resident* or consultant* or person*)) or clinician* or doctor* or physician* or general prac* or GP or primary care or nurs* or nutritionist* or dieti?ian or ((occupational or physical) adj therapist*) or pharmac*).ti,ab.	2,908,753
3	1 or 2	2,988,395
4	education, continuing/ or education, medical, continuing/ or education, nursing, continuing/ or education, professional, retraining/ or education, medical/ or education, nursing/ or education, public health professional/	155,183
5	((Education* or teach* or profession*) adj3 (intervention* or training* or curriculum* or course* or program* or workshop* or development* or certifica* or module*)).ti,ab.	192,846
6	4 or 5	329,567
7	overnutrition/ or overweight/ or obesity/ or Obesity Management/ or weight loss/ or bariatrics/	276,639
8	(((Obes* or overweight or weight or bariatric*) adj3 (manage* or control or loss)) or obes*).ti,ab.	515,231
9	7 or 8	569,223
10	Accreditation/ or certification/ or clinical competence/	140,189
11	(credential* or certific* or accredit* or knowledge* or skill* or competenc* or practice* or proficien*).ti,ab.	2,463,145
12	10 or 11	2,520,195
13	3 and 6 and 9 and 12	814
14	limit 13 to yr="2020 -Current"	259

Embase via OvidSP <1947 to present>

#	**Query**	**Results from 17th March 2025**
1	health care personnel/ or health practitioner/ or paramedical personnel/ or medical staff/ or nurse/ or nursing staff/ or dietitian/ or occupational therapist/ or pharmacist/ or physiotherapist/ or physician/	1,074,976
2	(((Health* or medic*) adj3 (professional* or staff* or worker* or provider* or trainee* or practitioner* or resident* or consultant* or person*)) or clinician* or doctor* or physician* or general prac* or GP or primary care or nurs* or nutritionist* or dieti?ian or ((occupational or physical) adj therapist*) or pharmac*).ti,ab.	4,077,318
3	1 or 2	4,376,020
4	continuing education/ or medical education/ or nursing education/	416,320
5	((Education* or teach* or profession*) adj3 (intervention* or training* or curriculum* or course* or program* or workshop* or development* or certifica* or module*)).ti,ab.	260,432
6	4 or 5	628,760
7	overnutrition/ or obesity/ or obesity management/ or body weight loss/ or bariatrics/	822,140
8	(((Obes* or overweight or weight or bariatric*) adj3 (manage* or control or loss)) or obes*).ti,ab.	795,353
9	7 or 8	1,052,903
10	accreditation/ or certification/ or clinical competence/	142,775
11	(credential* or certific* or accredit* or knowledge* or skill* or competenc* or practice* or proficien*).ti,ab.	3,225,479
12	10 or 11	3,283,501
13	3 and 6 and 9 and 12	2,110
14	limit 13 to yr="2020 -Current"	723

Web of Science

Searched in: Web of Science Core Collection, all editions (SCI-EXPANDED, SSCI, AHCI, CPCI-S, AHCI, CPCI-S, CPCI-SSH, BKCI-S, BKCI-SSH, ESCI, CCR-EXPANDED, IC)

#	**Query**	**Results from 10th March 2025**
1	(TI=(((Health* or medic*) near/3 (professional* or staff* or worker* or provider* or trainee* or practitioner* or resident* or consultant* or person*)) or clinician* or doctor* or physician* or general prac* or GP or primary care or nurs* or nutritionist* or dieti?ian or ((occupational or physical) near/1 therapist*) or pharmac*)) OR AB=(((Health* or medic*) near/3 (professional* or staff* or worker* or provider* or trainee* or practitioner* or resident* or consultant* or person*)) or clinician* or doctor* or physician* or general prac* or GP or primary care or nurs* or nutritionist* or dieti?ian or ((occupational or physical) near/1 therapist*) or pharmac*)	3,351,736
2	(TI=((Education* or teach* or profession*) near/3 (intervention* or training* or curriculum* or course* or program* or workshop* or development* or certifica* or module*))) OR AB=((Education* or teach* or profession*) near/3 (intervention* or training* or curriculum* or course* or program* or workshop* or development* or certifica* or module*))	495,088
3	(TI=(((Obes* or overweight or weight or bariatric*) near/3 (manage* or control or loss)) or obes*)) OR AB=(((Obes* or overweight or weight or bariatric*) near/3 (manage* or control or loss)) or obes*)	662,690
4	(TS=(credential* or certific* or accredit* or knowledge* or skill* or competenc* or practice* or proficien*)) OR AB=(credential* or certific* or accredit* or knowledge* or skill* or competenc* or practice* or proficien*)	5,361,677
5	#1 AND #2 AND #3 AND #4	858
6	#1 AND #2 AND #3 AND #4<br>Timespan: 2020-01-01 to 2025-03-10	321

ERIC

#	**Query**	**Results from 10th March 2025**
1	MAINSUBJECT.EXACT("Health Personnel") OR MAINSUBJECT.EXACT("Dietetics") OR MAINSUBJECT.EXACT("Allied Health Personnel") OR MAINSUBJECT.EXACT("Physicians") OR MAINSUBJECT.EXACT("Nurses")	16,409
2	abstract(((Health* OR medic*) NEAR/3 (professional* OR staff* OR worker* OR provider* OR trainee* OR practitioner* OR resident* OR consultant* OR person*)) OR clinician* OR doctor* OR physician* OR general prac* OR GP OR primary care OR nurs* OR nutritionist* OR dieti?ian OR ((occupational OR physical) NEAR/1 therapist*) OR pharmac*) OR title(((Health* OR medic*) NEAR/3 (professional* OR staff* OR worker* OR provider* OR trainee* OR practitioner* OR resident* OR consultant* OR person*)) OR clinician* OR doctor* OR physician* OR general prac* OR GP OR primary care OR nurs* OR nutritionist* OR dieti?ian OR ((occupational OR physical) NEAR/1 therapist*) OR pharmac*)	76,121
3	[S1] OR [S2]	82,349
4	MAINSUBJECT.EXACT("Competency Based Education") OR MAINSUBJECT.EXACT("Professional Development") OR MAINSUBJECT.EXACT("Continuing Education") OR MAINSUBJECT.EXACT("Medical Education")	51,699
5	abstract((Education* OR teach* OR profession*) NEAR/3 (intervention* OR training* OR curriculum* OR course* OR program* OR workshop* OR development* OR certifica* OR module*)) AND title((Education* OR teach* OR profession*) NEAR/3 (intervention* OR training* OR curriculum* OR course* OR program* OR workshop* OR development* OR certifica* OR module*))	45,245
6	[S4] OR [S5]	91,164
7	MAINSUBJECT.EXACT("Obesity")	3,182
8	abstract(((Obes* OR overweight OR weight OR bariatric*) NEAR/3 (manage* OR control OR loss)) OR obes*) AND title(((Obes* OR overweight OR weight OR bariatric*) NEAR/3 (manage* OR control OR loss)) OR obes*)	1,175

9	[S7] OR [S8]	3,342
10	MAINSUBJECT.EXACT("Accreditation (Institutions)") OR MAINSUBJECT.EXACT("Microcredentials")	6,989
11	abstract(credential* OR certific* OR accredit* OR knowledge* OR skill* OR competenc* OR practice* OR proficien*) AND title(credential* OR certific* OR accredit* OR knowledge* OR skill* OR competenc* OR practice* OR proficien*)	112,755
12	[S10] OR [S11]	117,415
13	[S3] OR [S6] AND [S9] AND [S12]	6
14	Limit 13 to yr="2020 -Current"	3

CINAHL

#	**Query**	**Results from 17th March 2025**
1	(MH "Health Personnel") OR (MH "Allied Health Personnel") OR (MH "Medical Staff") OR (MH "Nurses") OR (MH "Dietitians") OR (MH "Pharmacists") OR (MH "Physicians")	221,654
2	TI (((Health* or medic*) N3 (professional* or staff* or worker* or provider* or trainee* or practitioner* or resident* or consultant* or person*)) or clinician* or doctor* or physician* or general prac* or GP or primary care or nurs* or nutritionist* or dieti?ian or ((occupational or physical) adj therapist*) or pharmac*) OR AB (((Health* or medic*) N3 (professional* or staff* or worker* or provider* or trainee* or practitioner* or resident* or consultant* or person*)) or clinician* or doctor* or physician* or general prac* or GP or primary care or nurs* or nutritionist* or dieti?ian or ((occupational or physical) adj therapist*) or pharmac*)	1,320,122
3	S1 OR S2	1,409,865
4	(MH "Education, Continuing") OR (MH "Education, Medical, Continuing") OR (MH "Education, Nursing, Continuing") OR (MH "Education, Medical") OR (MH "Education, Nursing")	125,902
5	TI ((Education* or teach* or profession*) N3 (intervention* or training* or curriculum* or course* or program* or workshop* or development* or certifica* or module*)) OR AB ((Education* or teach* or profession*) N3 (intervention* or	132,923

	training* or curriculum* or course* or program* or workshop* or development* or certifica* or module*))	
6	S4 OR S5	241,289
7	(MH "Obesity") OR (MH "Overnutrition") OR (MH "Weight Loss")	114,331
8	TI (((Obes* or overweight or weight or bariatric*) N3 (manage* or control or loss)) or obes*) OR AB (((Obes* or overweight or weight or bariatric*) N3 (manage* or control or loss)) or obes*)	157,456
9	S7 OR S8	195,089
10	(MH "Accreditation") OR (MH "Certification") OR (MH "Clinical Competence")	78,090
11	TI (credential* or certific* or accredit* or knowledge* or skill* or competenc* or practice* or proficien*) OR AB (credential* or certific* or accredit* or knowledge* or skill* or competenc* or practice* or proficien*)	954,018
12	S10 OR S11	989,584
13	S3 AND S6 AND S9 AND S12	632
14	S3 AND S6 AND S9 AND S12<br>(Limiters: Publication Date: 20200101-20251231)	177

Google Scholar

First 200 results extracted

#	**Query**	**Results from 18th March 2025**
1	("healthcare professional*" OR "medical staff*" OR clinician* OR doctor* OR physician* OR nurs*) AND (education* OR training* or workshop*) AND ("obesity management*" OR obesity) AND (accredit* OR credential* OR competenc* OR knowledge* OR skill* OR certific*)	2,290

List of excluded studies with reasons

- Did not meet eligibility criteria for population (1-3)
- Did not meet eligibility criteria for intervention (4-11)

- Did not meet eligibility criteria for outcomes (12-15)
- Did not meet eligibility criteria for study type (16-47)

Supplementary Material S2: Characteristics and summary of included studies

Study details	Population and setting	Intervention, control	Outcomes measured	Data analysis, results	Author's conclusions and reviewer's comments
**Randomised controlled trials**					
**Authors:**<br>Agung et al.[24]<br><br>**Year published:**<br>2025<br><br>**Study design:**<br>RCT<br><br>**Country:**<br>Indonesia	**Sample size:**<br>N=64<br><br>**Inclusion criteria:**<br>"Healthcare professionals who had routinely provided adolescent health services at a primary health care centre for at least 6 months", only nurses and GPs were selected.<br><br>**Exclusion criteria:**<br>Midwives.<br><br>**Setting and population:**<br>GPs and nurses from primary healthcare centres, Indonesia.	**Intervention**<br>**Format and mode of delivery:**<br>• Self-paced online learning activities (including explanatory videos, audio recordings of exemplar consultations, quizzes, virtual interactive case vignettes, and written assignments)<br>• Online meetings (including group work, group presentations, interactive discussions led by a facilitator, and role-play exercises with adolescents)<br><br>**Topics covered:**<br>• Adolescent growth and development<br>• Parenting adolescents<br>• Healthy eating and physical activity<br>• Behavioural change counselling techniques for adolescent patients using MI approaches<br><br>**Duration and frequency:**<br>• Online learning activities: approximately 10 hours, to be completed in 4 weeks<br>• Four weekly online meetings, around 2 hours 30 minutes each (total 10 hours)<br><br>**Control**<br>Waitlist control.	**1. Knowledge**<br>• Knowledge of healthy eating, physical activity, screen time, basic communication skills with adolescent patients, psychosocial screening, MI techniques and parent roles in adolescent behaviour change<br><br>Knowledge was assessed using a 47-item adapted questionnaire, which generated a composite knowledge score.<br><br>**2. Counselling skills**<br>Counselling skills for weight management of adolescent patients:<br>• Introduction (greeting and opening the counselling session)<br>• Psychosocial screening (inviting the adolescent to talk without their parents)<br>• MI basic communication skills and MI stages<br>• Parental involvement (discussing parenting roles in supporting adolescent behaviour change)<br><br>Counselling skills were measured using audio-recorded counselling sessions and assessed by trained psychologists using a modified tool (Adolescent Behavioural Change-Counselling Assessment Tool, ABC-	**Data analysis**<br>Between-group comparisons used unpaired t-tests.<br><br>**1. Knowledge**<br>Pre-training knowledge value (mean ± SD)<br>• Control group (n=32): 63.7 ± 11.3<br>• Intervention group (n=31): 64.0 ± 9.9, p=0.902<br><br>Post-training knowledge value (mean ± SD)<br>• Control group (n=32): 67.2 ± 9.9<br>• Intervention group (n=31): 79.1 ± 12.3, p<0.001<br><br>**2. Counselling skills**<br>Pre-training counselling score (mean ± SD):<br>• Control group (n=32): 1.25 ± 0.54<br>• Intervention group (n=31): 1.43 ± 0.55, p=0.074<br><br>Post-training counselling score (mean ± SD)<br>• Control group (n=32): 1.49 ± 0.70<br>• Intervention group (n=31): 2.05 ± 0.42, p<0.001	**Author's conclusions:**<br>"This novel online training programme improved the knowledge and counselling skills of Indonesian primary healthcare professionals. Greater emphasis on engaging parents and more guidance on conducting telehealth counselling may improve parental involvement in future iterations."<br><br>**Reviewer's comments:**<br>This study found that an online training programme significantly improved healthcare professionals' knowledge and counselling skills for adolescent weight management. Post-training, the intervention group's knowledge score was significantly higher than the control, and counselling scores also showed significant improvement.

<table>
<tr><td></td><td></td><td></td><td>CAT) which used a Likert scale (0 = no statements heard, 3 = all statements heard).</td><td></td><td>This study has clearly met 7/12 (58%) criteria in the critical appraisal tool.</td></tr>
<tr><td>Authors:
Allison et al.[19]

Year published:
2023

Study design:
RCT

Country:
Australia</td><td>Sample size:
N=80

Inclusion criteria:
“Current registration with the Australian Health Practitioner Regulation Agency, access to a computer with internet connection, willingness to complete ~8 hours of online education, and willingness to refrain from participate in any weight management professional development activity external to the study for the intervention period.”

Exclusion criteria:
None.

Setting and population:
Physical therapists working in Australia.</td><td>Intervention
Format and mode of delivery:
• Six self-directed online modules, featuring quizzes, lectures, case studies, and expert responses (including case and video exemplars of clinical interactions, patient feedback from consultations, strategies to make patients feel more comfortable discussing weight, and resources for therapists and patients)

Topics covered:
• Overweight, obesity and OA
• Overview of weight regulation, overweight, and obesity
• Examining weight stigma and personal beliefs about weight
• Communication approaches for addressing weight management
• Interventions for weight management
• Health behaviour change support

Duration and frequency:
• Participants were given 6 weeks to complete the 6 modules
• Participants took 6.9 ± 2.3 hours (mean ± SD) to complete all modules

Control
No intervention.</td><td>1. Confidence
• Self-reported confidence in knowledge about weight management for patients with OA
• Self-reported confidence in clinical skills for weight management for patients with OA

Confidence was measured using a questionnaire developed specifically for the study (higher scores = higher confidence).

2. Competence
• Self-perceived competence in nutrition care to patients with chronic disease

Self-efficacy was measured using the nutrition competence (NUTCOMP) questionnaire (higher scores = higher perceived competence).

3. Attitudes
• Weight-stigmatised attitudes (including dislike, fear of fat, and willpower)

Attitudes were measured using the Antifat Attitudes Questionnaire (AFA, higher scores = more stigmatised attitudes).</td><td>Data analysis
Linear regression, adjusted for baseline and stratifying variables, estimated the mean difference in change (95% CI) between groups for outcomes.

1. Confidence
Confidence in knowledge (mean ± SD):
Change post-intervention
• Control group (n=41): 2.8 ± 6.1
• Intervention group (n=38): 25.4 ± 7.6
• Adjusted mean difference: 22.6 (19.6, 25.5)

Confidence in clinical skills (mean ± SD):
Change post-intervention
• Control group (n=41): 0.0 ± 4.3
• Intervention group (n=38): 14.9 ± 5.0
• Adjusted mean difference: 14.6 (12.7, 16.4)

2. Competence
Competence in nutrition care (mean ± SD):
Change post-intervention
• Control group (n=41): -0.4 ± 9.1
• Intervention group (n=38): 39.5 ± 15.4
• Adjusted mean difference: 39.9 (34.5, 45.4)

3. Attitudes
Weight-stigmatised attitudes (mean ± SD):
AFA dislike subscale, change post-intervention
• Control group (n=41): 0.8 ± 6.4
• Intervention group (n=38): -2.3 ± 6.8
• Adjusted mean difference: -3.1 (-5.9, -0.3)

AFA fear subscale, change post-intervention
• Control group (n=41): -0.7 ± 4.0
• Intervention group (n=38): -0.1 ± 5.1
• Adjusted mean difference: 0.3 (-1.6, 2.2)</td><td>Author’s conclusions:
“An online education program improves physical therapists’ short-term confidence in knowledge and skills in weight management for people with osteoarthritis and reduces weight-stigmatized attitudes.”

Reviewer’s comments:
This study found that a six-week, self-directed online module programme significantly enhanced healthcare professionals' confidence in obesity knowledge and clinical skills for managing overweight/obesity in patients with OA. It also significantly improved their perceived competence in nutrition care. Furthermore, the programme significantly reduced weight-stigmatising attitudes, specifically concerning dislike and willpower subscales, compared to the control group.

This study has clearly met 8/9 (89%) criteria in the critical appraisal tool.</td></tr>
</table>

				AFA willpower subscale, change post-intervention<br>• Control group (n=41): -0.6 ± 3.6<br>• Intervention group (n=38): -5.0 ± 4.7<br>• Adjusted mean difference: -4.2 (-6.0, -2.5)	
**Authors:**<br>Isom et al.[18]<br><br>**Year published:**<br>2020<br><br>**Study design:**<br>RCT<br><br>**Country:**<br>United States	**Sample size:**<br>N=54<br><br>**Inclusion criteria:**<br>• Dietetic interns participating in a dietetic internship obesity class day<br><br>**Exclusion criteria:**<br>• Licensed or practicing dietitians<br>• Dietetic interns who assisted in study development<br><br>**Setting and population:**<br>Dietetic interns participating in a dietetic internship class day, Boston	**Intervention**<br>**Format and mode of delivery:**<br>• Educational video in addition to standard didactic obesity training (lectures)<br>• Post-video discussion<br><br>**Topics covered:**<br>• Educational video: contributing factors to obesity, prevalence and impact of bias, strategies to reduce weight stigma, practical guidance for healthcare professionals<br>• Post-video discussion: experiences working with patients with obesity<br>• Didactic obesity training: medical and surgical weight management, nutritional care of patients with obesity<br><br>**Duration and frequency:**<br>• Video: 17-minutes<br>• Post-video discussion: "brief"<br>• Standard didactic obesity training: 6 hours total<br><br>**Control**<br>Standard didactic obesity training only.	**1. Obesity Knowledge**<br>• Beliefs about the controllability of obesity<br>• Knowledge of obesity and associated health risks<br><br>Obesity knowledge was measured using the 8-items Beliefs About Obese People (BAOP, higher scores = stronger belief that obesity is not under the control of a person with obesity) and the Obesity Risk Knowledge-10 (ORK-10, maximum score = 10).<br><br>**2. Bias**<br>• Explicit weight bias<br>• Implicit weight bias<br><br>Explicit weight bias was measured using the Anti-Fat Attitudes questionnaire (AFA, higher scores = more stigmatised attitudes). Implicit weight bias was measured using the Implicit Association Test (higher scores = greater implicit weight bias)	**Data analysis**<br>Descriptive statistics. To assess score changes, independent t-tests (parametric) or Mann-Whitney U tests (nonparametric) were conducted on change scores from baseline to post-education and post-education to one-month.<br><br>**1. Obesity knowledge**<br>BAOP score (mean ± SD)<br>Control group<br>• Baseline (n=24): 21.8 ± 3.0<br>• Post-intervention (n=24): 22.7 ± 3.6, p=0.18<br>• 1-month (n=19): 21.4 ± 3.6, p=0.006<br>Intervention group<br>• Baseline (n=27): 23.5 ± 4.6<br>• Post-intervention (n=26): 23.7 ± 4.0, p=0.90<br>• 1-month (n=20): 23.0 ± 4.6, p=1.00<br>Between-group differences<br>• Baseline-post-intervention difference: p=0.49<br>• Post-intervention-1-month difference: p=0.24<br><br>ORK-10 score (mean ± SD)<br>Control group<br>• Baseline (n=24): 6.7 ± 1.9<br>• Post-intervention (n=24): 6.9 ± 1.6, p=0.32<br>• 1-month (n=19): 6.6 ± 1.7, p=0.46<br>Intervention group<br>• Baseline (n=27): 6.7 ± 1.7<br>• Post-intervention (n=26): 6.9 ± 1.2, p=0.87<br>• 1-month (n=20): 6.2 ± 1.6, p=0.10<br>Between-group differences<br>• Baseline-post-intervention difference: p=0.62<br>• Post-intervention-1-month difference: p=0.52<br><br>**2. Bias**<br>Explicit weight bias, AFA total score (mean ± SD)<br>Control group<br>• Baseline (n=24): 38.7 ± 17.6	**Author's conclusions:**<br>"A brief educational video intervention caused a significant decrease in weight bias among dietetic interns. Therefore, further research interventions are needed to sustain a decreased level of bias."<br><br>**Reviewer's comments:**<br>This study found that an educational video combined with didactic training significantly reduced healthcare professionals' explicit and implicit weight bias immediately post-intervention. However, this intervention did not significantly improve general obesity knowledge or beliefs about obesity controllability compared to didactic training alone.<br><br>This study has clearly met 8/10 (80%) criteria in the critical appraisal tool.

				• Post-intervention (n=24): 39.8 ± 20.5, p=0.72<br>• 1-month (n=19): 38.3 ± 18.1, p=0.72<br>Intervention group:<br>• Baseline (n=27): 30.4 ± 12.0<br>• Post-intervention (n=26): 25.8 ± 11.1, p=0.005<br>• 1-month (n=20): 32.1 ± 13.6, p=0.001<br>Between-group<br>• Baseline-post-intervention difference: p=0.03<br>• Post-intervention-1-month difference: p=0.037<br><br>AFA dislike subscale (mean ± SD)<br>Control group<br>• Baseline (n=24): 1.8 ± 1.5<br>• Post-intervention (n=24): 2.0 ± 1.8, p=0.36<br>• 1-month (n=19): 1.8 ± 1.3, p=1.00<br>Intervention group:<br>• Baseline (n=27): 1.2 ± 1.0<br>• Post-intervention (n=26): 1.0 ± 0.9, p=0.045<br>• 1-month (n=20): 1.3 ± 1.1, p=0.07<br>Between-group<br>• Baseline-post-intervention difference: p=0.034<br>• Post-intervention-1-month difference: p=0.19<br><br>AFA fear of fat subscale (mean ± SD)<br>Control group<br>• Baseline (n=24): 4.9 ± 2.5<br>• Post-intervention (n=24): 5.0 ± 2.6, p=0.72<br>• 1-month (n=19): 5.0 ± 2.5, p=0.85<br>Intervention group:<br>• Baseline (n=27): 4.3 ± 1.9<br>• Post-intervention (n=26): 3.8 ± 1.8, p=0.057<br>• 1-month (n=20): 4.4 ± 2.0, p=0.016<br>Between-group<br>• Baseline-post-intervention difference: p=0.22<br>• Post-intervention-1-month difference: p=0.06<br><br>AFA willpower subscale (mean ± SD)<br>Control group<br>• Baseline (n=24): 3.9 ± 1.7<br>• Post-intervention (n=24): 3.6 ± 2.1, p=0.36<br>• 1-month (n=19): 3.7 ± 2.1, p=0.19<br>Intervention group	

				• Baseline (n=27): 2.9 ± 1.4<br>• Post-intervention (n=26): 2.6 ± 1.4, p=0.17<br>• 1-month (n=20): 3.2 ± 1.6, p=0.027<br>Between-group<br>• Baseline-post-intervention difference: p=1.00<br>• Post-intervention-1-month difference: p=0.39<br><br>Implicit weight bias score (mean ± SD)<br>Control group<br>• Baseline (n=23): 5.3 ± 1.7<br>• Post-intervention (n=23): 5.4 ± 1.5, p=0.604<br>• 1-month (n=19): 4.7 ± 1.2, p=0.08<br>Intervention group<br>• Baseline (n=27): 5.6 ± 1.5<br>• Post-intervention (n=26): 4.3 ± 1.1, p=0.002<br>• 1-month (n=20): 4.2 ± 1.9, p=0.95<br>Between-group<br>• Baseline-post-intervention difference: p=0.003<br>• Post-intervention-1-month difference: p=0.29	
**Authors:**<br>Sherf-Dagan et al.[27]<br><br>**Year published:**<br>2022<br><br>**Study design:**<br>RCT<br><br>**Country:**<br>Israel	**Sample size:**<br>N=506[a]<br><br>**Inclusion criteria:**<br>• Employees of a chain of private medical centres (including hospitals, medical centres with operating rooms and outpatient care)<br><br>**Exclusion criteria:**<br>• Not stated<br><br>**Setting and population:**<br>Employees of a chain of private medical centres, Israel.	**Intervention**<br>**Format and mode of delivery:**<br>• Online module, including a short quiz<br><br>**Topics covered:**<br>• Knowledge about obesity (including obesity definition and prevalence, risk factors, and treatment options)<br>• Weight bias, stigma, and discrimination definitions and impact<br>• Strategies to reduce weight bias, stigma, and discrimination<br><br>**Duration and frequency:**<br>• One 15-minute module<br><br>**Control**<br>No intervention.	**1. Attitudes**<br>• Explicit anti-fat attitudes (dislike, fear about fat, willpower)<br>• Fat-phobic attitudes towards people with obesity<br><br>Attitudes were measured using the Anti-Fat Attitudes (AFA) questionnaire and short form fat-phobia scale (higher scores = stronger anti-fat attitudes).<br><br>**2. Beliefs**<br>• Beliefs about the causes of obesity[c]<br><br>Beliefs were measured using an adapted version of the beliefs about the causes of obesity questionnaire, which uses a 5-point Likert scale (1=not at all important, 5=extremely important).	**Data analysis**<br>Linear mixed models for repeated measures assessed continuous variable changes within and between groups over time. The Cochran Q test examined dichotomous variable changes within groups across time. Intention-to-treat analysis was employed for study endpoints.<br><br>**1. Attitudes**<br>AFA total score (mean)<br>Control group<br>• Baseline (n=255): 3.27<br>• 7-days (n=118): 3.01, p≤0.05<br>• 30-days (n=72): 3.03, p≤0.05<br>Intervention group<br>• Baseline (n=249): 3.17<br>• 7-days (n=112): 2.94, p≤0.05<br>• 30-days (n=73): 2.96, p≤0.05<br>Between-group: p=0.892<br><br>AFA dislike subscale (mean)<br>Control group<br>• Baseline (n=255): 1.29<br>• 7-days (n=118): 1.26	**Author's conclusions:**<br>"A single exposure to an online education module on weight bias and knowledge about obesity may confer only a modest short-term improvement in medical centre employees' fat-phobic attitudes toward people with obesity. Future studies should examine if reexposure to such intervention could impact weight bias, stigma, and discrimination among medical centre staff in the long-term."<br><br>**Reviewer's comments:**<br>This study found that a 15-minute online module on obesity, weight bias,

				• 30-days (n=72): 1.35<br>Intervention group<br>• Baseline (n=249): 1.13<br>• 7-days (n=112): 1.21<br>• 30-days (n=73): 1.22<br>Between-group: p=0.523<br><br>AFA fear of fat subscale (mean)<br>Control group<br>• Baseline (n=255): 6.12<br>• 7-days (n=118): 5.62, p≤0.05<br>• 30-days (n=72): 5.27, p≤0.05<br>Intervention group<br>• Baseline (n=249): 6.15<br>• 7-days (n=112): 5.74, p≤0.05<br>• 30-days (n=73): 5.66, p≤0.05<br>Between-group: p=0.328<br><br>AFA willpower subscale (mean)<br>Control group<br>• Baseline (n=255): 5.05<br>• 7-days (n=118): 4.45, p≤0.05<br>• 30-days (n=72): 4.71<br>Intervention group<br>• Baseline (n=249): 4.95<br>• 7-days (n=112): 4.17, p≤0.05<br>• 30-days (n=73): 4.27, p≤0.05<br>Between-group: p=0.430<br><br>Short form fat-phobia scale (mean)<br>Control group<br>• Baseline (n=241): 3.41<br>• 7-days (n=113): 3.40<br>• 30-days (n=71): 3.37<br>Intervention group<br>• Baseline (n=232): 3.45<br>• 7-days (n=106): 3.36, p≤0.05<br>• 30-days (n=72): 3.33, p≤0.05<br>Between-group: not significant<br><br>**2. Beliefs**<br>Contribution of overeating to obesity (mean)	and stigma did not significantly impact healthcare professionals' anti-fat attitudes or beliefs about obesity causes compared to a control. While both groups showed slight reductions in some anti-fat attitude subscales, between-group differences were not statistically significant.<br><br>This study has clearly met 6/9 (67%) criteria in the critical appraisal tool.

				Control group<br>• Baseline (n=231): 4.69<br>• 7-days (n=109): 4.59<br>• 30-days (n=69): 4.62<br>Intervention group<br>• Baseline (n=222): 4.74<br>• 7-days (n=102): 4.63<br>• 30-days (n=72): 4.66<br>Between group: p=0.988<br><br>Contribution of lack of willpower to obesity<br>Control group<br>• Baseline (n=231): 4.12<br>• 7-days (n=109): 4.04<br>• 30-days (n=69): 4.00<br>Intervention group<br>• Baseline (n=222): 4.12<br>• 7-days (n=102): 3.94<br>• 30-days (n=72): 3.91<br>Between group: p=0.619<br><br>Contribution of sedentary lifestyle to obesity<br>Control group<br>• Baseline (n=231): 4.14<br>• 7-days (n=109): 3.95<br>• 30-days (n=69): 4.12<br>Intervention group<br>• Baseline (n=222): 4.26<br>• 7-days (n=102): 4.12<br>• 30-days (n=72): 4.08<br>Between group: p=0.274	
**Quasi-experimental studies**					
**Authors:**<br>Baillargeon et al.[22]<br><br>**Year published:**<br>2020<br><br>**Study design:**	**Sample size:**<br>N = 35<br><br>**Inclusion criteria:**<br>"A family medicine group was eligible to participate if at least one primary care physician and one nurse were interested."	**Intervention**<br>**Format and mode of delivery:**<br>• In-person preceptorship (including interactive sessions with content experts, case discussions, and observation of real-patient encounters with an interdisciplinary obesity management team)	**1. Attitudes**<br>• Self-rated attitude towards patients with obesity<br><br>**2. Confidence**<br>• Self-rated confidence in obesity management<br><br>**3. Self-efficacy**	**Data analysis**<br>Changes over time (baseline, 1 month, 1 year) were analysed using mixed-model ANOVA with Tukey-Kramer post-hoc tests. Baseline-to-1-year changes in clinical vignettes and patient records were assessed using Wilcoxon and Fisher exact tests, respectively.<br><br>**1. Attitudes**<br>Attitude towards patients with obesity (mean):<br>Physicians	**Author's conclusions:**<br>"A multimodal educational intervention for obesity management can improve PCPs' attitude and self-efficacy for obesity management and lifestyle counselling. This translates into beneficial practice

Before-and-after study<br><br>**Country:**<br>Canada	**Exclusion criteria:**<br>Not stated.<br><br>**Setting and population:**<br>Physicians and nurses in Family medicine groups, Quebec.	• Online platform (including a discussion forum and literature/tools for obesity management)<br>• Webinars<br><br>**Topics covered:**<br>• Preceptorship: initial patient evaluation, medical and surgical treatment options, behavioural approach to lifestyle modification, practical tips for diet and physical activity counselling, paediatric obesity, nutritional approach, physical activity interventions<br>• Webinars: nutrition, physical activity, behavioural approaches to lifestyle modification<br><br>**Duration and frequency:**<br>• Preceptorship: 2 days (19 hours total)<br>• Discussion forum: ongoing for one year<br>• Webinars: 6 total, 30-45 minutes per session, held monthly<br><br>**Control**<br>Not applicable.	• Perceived self-efficacy in physical activity and nutritional counselling<br><br>Attitudes, confidence, and self-efficacy were measured using self-administered 100 mm visual-analog scales (1mm = negative attitude/not confident; 100m = positive attitude/very confident).<br><br>**4. Practice changes**<br>Self-reported practice changes for patient consulting, specifically for obesity:<br>• Evaluation of readiness to change lifestyle<br>• Offer of weight management follow-up<br>• Offer of group seminars<br>• Use of food diary<br>• Referral to a dietitian<br>• Use of pedometer<br>• Offer/discuss antiobesity drugs<br><br>Practice changes were measured via reposes to clinical vignettes via a Likert scale (1 = never, 5 = always) and review of electronic patient charts.<br><br>**5. Patient outcomes**<br>• Change in waist circumference<br>• Weight loss<br><br>Patient outcomes were measured by review of electronic patient charts.	• Baseline: 61 mm<br>• 1 month: 77 mm, $p<0.05$<br>• 1 year: 85 mm, $p<0.001$<br>Nurses<br>• No significant change throughout the study period.<br><br>**2. Confidence**<br>Confidence in managing obesity (mean):<br>• Baseline: 48 mm<br>• 1 month: 72 mm<br>• 1 year: maintained, $p<0.05$<br><br>Confidence in helping adolescents/children manage weight (mean):<br>Physicians<br>• Baseline: 34 mm<br>• 1 month: 54 mm, $p<0.05$<br>• 1 year: 60 mm, $p<0.05$<br>Nurses<br>• Baseline: 30 mm<br>• 1 month: 42 mm, not significant<br>• 1 year: 60 mm, $p<0.05$<br><br>**3. Self-efficacy**<br>Self-efficacy to give nutritional advice (mean):<br>• Baseline: 48mm<br>• 1 month: 74 mm, $p<0.05$<br>• 1 year: 72 mm, $p<0.05$<br><br>Self-efficacy to give advice on physical activity (mean):<br>• Baseline: 54mm<br>• 1 month: 72mm, $p<0.05$<br>• 1 year: 77 mm, $p<0.05$<br><br>Self-efficacy to prescribe anti-obesity drugs (mean):<br>Physicians<br>• Baseline: 44 mm<br>• 1 month: 66 mm, $p<0.05$<br>• 1 year: maintained, $p<0.05$<br><br>**4. Practice changes**	changes and patient-related outcomes."<br><br>**Reviewer's comments:**<br>This study found that a mixed-mode obesity education programme significantly improved physicians' attitudes, confidence, and self-efficacy in obesity management, alongside marked increases in specific self-reported and documented practice changes. It also found improvements in practices and patient outcomes, including weight loss and reduced waist circumference.<br><br>This study has clearly met 4/7 (57%) criteria in the critical appraisal tool.

				Self-reported practice changes (median (IQR)):<br>Evaluation of readiness to change lifestyle<br>• Baseline: 4.00 (1.00); 1 year: 5.00 (1.00), p<0.05<br><br>Offer of weight management follow-up<br>• Baseline: 4.25 (1.25); 1 year: 5.00 (0.50), p<0.05<br><br>Offer of group seminars<br>• Baseline: 3.00 (3.00); 1 year: 1.00 (2.00), not significant<br><br>Use of a food diary<br>• Baseline: 2.00 (2.00); 1 year: 4.00 (1.75), p<0.05<br><br>Referral to a dietitian<br>• Baseline: 5.00 (1.00); 1 year: 5.00 (1.00), not significant<br><br>Use of pedometer<br>• Baseline: 3.00 (3.00); 1 year: 4.00 (1.00), p<0.05<br><br>Offer/discuss anti-obesity drugs<br>• Baseline: 3.00 (2.00); 1 year: 2.00 (2.00), not significant<br><br>Chart review practice changes (% of charts, n = 53):<br>Evaluation of readiness to change lifestyle<br>• Baseline: 12.7%; 1 year: 32.1%, p<0.05<br><br>Weight measurement<br>• Baseline: 63.6%; 1 year: 83.0%, p<0.05<br><br>Waist circumference measurement<br>• Baseline: 7.3%; 1 year: 35.8%, p<0.0001<br><br>Blood pressure measurement<br>• Baseline: 74.5%; 1 year: 84.9%<br><br>Medication listing<br>• Baseline: 72.7%; 1 year: 52.8%<br><br>**5. Patient outcomes**	

				Change in waist circumference (mean)<br>• 1 year: -4.3 cm, p<0.0001<br><br>Weight loss (mean)<br>• 1 year: 4.5 kg, p<0.0001	
**Authors:**<br>Bernard-Genest et al.[17]<br><br>**Year published:**<br>2020<br><br>**Study design:**<br>Before-and-after study<br><br>**Country:**<br>Canada	**Sample size:**<br>N = 70<br><br>**Inclusion criteria:**<br>"Physicians, nurses, nurse practitioners, dieticians, physiotherapists, occupational therapists, psychologists, social workers, and trainees from these professions who work with children."<br><br>**Exclusion criteria:**<br>Not stated.<br><br>**Setting and population:**<br>Health professionals and trainees who work with children, Toronto.	**Intervention**<br>**Format and mode of delivery:**<br>• White board-style cartoon video, narrated from the perspective of a 15-year-old female<br><br>**Topics covered:**<br>• Young person's perspective on weight-related conversations<br>• Preferred timing and approach to discussions<br>• Strengths-based, health-focused language<br>• Collaborative goal setting<br>• Preference for inclusive, non-restrictive strategies<br><br>**Duration and frequency:**<br>• One 6-minute video<br><br>**Control**<br>Not applicable.	**1. Self-efficacy**<br>• Perceived self-efficacy in initiating a conversation with a family or paediatric patient about overweight<br><br>Self-efficacy was measured using an adapted questionnaire with a Likert-type scale (0 = not confident, 100 = very confident).	**Data analysis**<br>Pre-, post-, and 6-month retention self-efficacy scores were analysed using the Friedman test, followed by Wilcoxon signed-rank tests for paired comparisons.<br><br>**1. Self-efficacy**<br>Confidence in initiating overweight conversation with a family (mean ± SD, n = 39):<br>• Baseline: 66.2 ± 28.7<br>• Immediately after intervention: 75.8 ± 26.0, p<0.001<br>• 6 months: 72.4 ± 22.5, p=0.129	**Author's conclusions:**<br>"The present study suggests educational videos may be an effective tool for facilitating healthy weight-related conversations between HCPs and their paediatric patients."<br><br>**Reviewer's comments:**<br>This study found that a brief 6-minute video from a young person's perspective significantly enhanced healthcare professionals' self-efficacy in initiating overweight-related conversations with paediatric patients. While a modest decline was observed at 6-month follow-up, this difference from immediate post-intervention was not statistically significant.<br><br>This study has clearly met 5/7 (71%) criteria in the critical appraisal tool.
**Authors:**<br>Bonder et al.[30]	**Sample size:**<br>N=9<br><br>**Inclusion criteria:**	**Intervention**<br>**Format and mode of delivery:**<br>• In-person or online workshop, featuring a knowledge translation casebook (included simulation	**1. Self-efficacy**<br>Self-perceived confidence in:<br>• Having positive weight-related conversations with clients	**Data analysis**<br>Descriptive statistics only.<br><br>**1. Self-efficacy**	**Author's conclusions:**<br>"The Casebook was acceptable and appeared to increase self-efficacy about having weight-

**Year published:**<br>2020<br><br>**Study design:**<br>Before-and-after study<br><br>**Country:**<br>Canada	• Clinicians providing care to children in a healthcare setting<br>• Have self-reported experience discussing weight-management with children with ASD<br>• Able to speak and read in English<br><br>**Exclusion criteria:**<br>Not stated.<br><br>**Setting and population:**<br>Clinicians (paediatricians, nurses, and dietitians) working at a large paediatric teaching hospital and clinicians participating in a distance learning programme, Ontario.	scenarios that reflected common parent experiences discussing weight gain in children with ASD, including challenges such as medication-related weight gain)<br><br>**Topics covered:**<br>• Having positive weight-related conversations with children and parents<br><br>**Duration and frequency:**<br>• One 2-hour workshop<br><br>**Control**<br>Not applicable.	• Identifying who should be involved in having weight-related conversations<br>• Identifying appropriate times to have a weight-related conversation<br>• Ability to enhance family engagement<br>• Using specific communication techniques<br><br>Self-efficacy was measured using a Likert scale (1 = not confident, 5 = very confident).<br><br>**2. Clinical skills**<br>• Explore child's willingness/ability to discuss weight<br>• Acknowledge barriers to healthy lifestyle changes<br>• Use growth-chart or other tools to assess weight trajectory<br>• Celebrate weight-related successes<br>• Collaborate with the family<br>• Create goals that are important to the child<br><br>Clinical skills were measured using a Likert scale (1 = never, 5 = all the time).	Having positive weight-related conversations with clients (mean, n=9)<br>• Pre-workshop: 2.4; 2 months: 2.9<br><br>Identifying who should be involved in having weight-related conversations (mean, n=9)<br>• Pre-workshop: 2.9; 2 months: 3.7<br><br>Identifying appropriate times to have a weight-related conversation (mean, n=9)<br>• Pre-workshop: 2.4; 2 months: 3.3<br><br>Ability to enhance family engagement (mean, n=9)<br>• Pre-workshop: 2; 2 months: 3.1<br><br>Using specific communication techniques (mean, n=9)<br>• Pre-workshop: 1.8; 2 months: 3.0<br><br>**2. Clinical skills**<br>Explore child's willingness/ability to discuss weight (mean, n=9)<br>• Pre-workshop: 3; 2 months: 3.4<br><br>Acknowledge barriers to healthy lifestyle changes (mean, n=9)<br>• Pre-workshop: 3.8; 2 months: 3.9<br><br>Use growth-charts or other tools to assess weight trajectory (mean, n=9)<br>• Pre-workshop: 3.2; 2 months: 3.2<br><br>Celebrate weight-related successes (mean, n=9)<br>• Pre-workshop: 3.8; 2 months: 3.9<br><br>Collaborate with the family (mean, n=9)<br>• Pre-workshop: 3.9; 2 months: 3.9<br><br>Create goals that are important to the child (mean, n=9)<br>• Pre-workshop: 3.4; 2 months: 3.4	related conversations with children with ASD and parents. More robust implementation strategies are needed to foster the uptake of best practices in weight-related conversations into clinical practice."<br><br>**Reviewer's comments:**<br>This study found that a two-hour workshop enhanced healthcare professionals' self-efficacy in conducting positive weight-related conversations, identifying key participants and appropriate timing, improving family engagement, and utilizing specific communication techniques. Some improvements were seen in clinical skills, particularly exploring a child's willingness or ability to discuss weight.<br><br>This study has clearly met 5/7 (71%) criteria in the critical appraisal tool.
**Authors:**<br>Chae et al.[20]	**Sample size:**<br>N=35	**Intervention**<br>**Format and mode of delivery:**	**1. Self-efficacy**<br>Confidence in performing elements of obesity care:	**Data analysis**<br>To assess within-group changes, paired t-tests were used for obesity care self-efficacy and clinical practice	**Author's conclusions:**<br>"Our OM curriculum is feasible with IM residents

**Year published:**<br>2024<br><br>**Study design:**<br>Non-randomised controlled trial<br><br>**Country:**<br>United States	**Inclusion criteria:**<br>• Internal Medicine residents in one of two residency programmes<br>• Have at least two primary care clinic sessions both before and after the planned intervention period<br><br>**Exclusion criteria:**<br>• Internal Medicine-Paediatrics programme residents<br><br>**Setting and population:**<br>Internal Medicine residents from two residency programmes affiliated with the same institution in Baltimore.	• Seven self-paced online modules (including e-lectures and knowledge checks)<br>• Three live case-based discussions (in-person and online)<br>• Optional participation<br><br>**Topics covered:**<br>• Lifestyle counselling (including nutrition, physical activity, behaviour change)<br>• Medication management (including weight-gain-promoting medications, anti-obesity medications)<br>• Bariatric procedures (including bariatric surgery, management of post-bariatric surgery patients)<br>• Healthcare experiences for patients with obesity (including weight bias/stigma)<br><br>**Duration and frequency:**<br>• Modules: 15-minutes each (1 hour 45 minutes total), to be completed within 3 months<br>• Live case-based discussions: monthly sessions (average 30-minutes per session, 3 sessions total)<br><br>**Control**<br>No intervention.	• Nutritional counselling<br>• Physical activity counselling<br>• Behaviour change counselling<br>• Identifying weight-gain-promoting medications<br>• Discussing anti-obesity medications<br>• Discussing bariatric surgery<br>• Managing post-bariatric surgical patients in the primary care setting<br><br>Self-efficacy was measured using a Likert scale (1 = not confident, 4 = very confident).<br><br>**2. Clinical practice habits**<br>Self-reported frequency in performing elements of obesity care:<br>• Nutritional counselling<br>• Physical activity counselling<br>• Behaviour change counselling<br>• Identifying weight-gain-promoting medications<br>• Discussing anti-obesity medications<br>• Discussing bariatric surgery<br>• Managing post-bariatric surgical patients in the primary care setting<br><br>Clinical practice habits was measured using a Likert scale (1 = never, 4 = always).	habits. Between intervention and control groups, a differences-in-differences analysis using unpaired t-tests compared mean changes from baseline to 6-month surveys.<br><br>**1. Self-efficacy**<br>Nutrition counselling (mean ± SD)<br>Control group (n=18)<br>• Baseline: 2.9 ± 0.6; 6 months: 3.1 ± 0.2, p=0.27<br>Intervention group (n=17)<br>• Baseline: 2.6 ± 0.1; 6 months: 3.5 ± 0.6, p<0.01<br>Between-group: p=0.02<br><br>Physical activity counselling (mean ± SD)<br>Control group<br>• Baseline: 3.0 ± 0.6; 6 months: 3.1 ± 0.5, p=0.43<br>Intervention group<br>• Baseline: 2.8 ± 0.1; 6 months: 3.4 ± 0.7, p=0.01<br>Between-group: p=0.06<br><br>Behaviour change counselling (mean ± SD)<br>Control group<br>• Baseline: 2.6 ± 0.6; 6 months: 2.9 ± 0.5, p<0.01<br>Intervention group<br>• Baseline: 2.3 ± 0.1; 6 months: 3.5 ± 0.6, p<0.01<br>Between-group: p<0.01<br><br>Identifying weight-gain-promoting medications (mean ± SD)<br>Control group<br>• Baseline: 2.9 ± 0.8; 6 months: 2.9 ± 0.7, p=0.79<br>Intervention group<br>• Baseline: 2.4 ± 0.2; 6 months: 3.2 ± 0.8, p<0.01<br>Between-group: p=0.01<br><br>Discussing anti-obesity medications (mean ± SD)<br>Control group<br>• Baseline: 1.8 ± 0.9; 6 months: 2.3 ± 0.8, p=0.07<br>Intervention group<br>• Baseline: 1.4 ± 0.1; 6 months: 2.5 ± 0.7, p<0.01<br>Between-group: p=0.03	and increases residents' obesity care self-efficacy beyond what is achieved with usual IM training."<br><br>**Reviewer's comments:**<br>This study found that a combined online module and live discussion programme significantly improved healthcare professionals' self-efficacy in various aspects of obesity care, including nutritional, physical activity, and behaviour change counselling, and managing obesity medications and bariatric surgery patients. The intervention group also showed significant improvements in clinical practice habits related to anti-obesity medication discussions and bariatric surgery.<br><br>This study has clearly met 6/9 (67%) criteria in the critical appraisal tool.

				Discussing bariatric surgery (mean ± SD)<br>Control group<br>• Baseline: 2.4 ± 0.7; 6 months: 2.8 ± 0.4, p=0.05<br>Intervention group<br>• Baseline: 2.0 ± 0.2; 6 months: 2.9 ± 0.7, p<0.01<br>Between-group: p=0.03<br><br>Post-op management (mean ± SD)<br>Control group<br>• Baseline: 2.2 ± 0.7; 6 months: 2.3 ± 0.6, p=0.38<br>Intervention group<br>• Baseline: 2.0 ± 0.1; 6 months: 2.6 ± 0.6, p<0.01<br>Between-group: p=0.05<br><br>**2. Clinical practice habits**<br>Nutrition counselling (mean ± SD)<br>Control group<br>• Baseline: 3.7 ± 0.7; 6 months: 4.1 ± 0.6, p=0.01<br>Intervention group<br>• Baseline: 3.7 ± 0.2; 6 months: 4.0 ± 0.5, p=0.21<br><br>Physical activity counselling (mean ± SD)<br>Control group<br>• Baseline: 3.7 ± 0.7; 6 months: 4.2 ± 0.6, p<0.01<br>Intervention group<br>• Baseline: 3.6 ± 0.1; 6 months: 3.8 ± 0.5, p=0.38<br><br>Behaviour change counselling (mean ± SD)<br>Control group<br>• Baseline: 2.7 ± 0.9; 6 months: 3.4 ± 0.9, p<0.01<br>Intervention group<br>• Baseline: 2.9 ± 0.2; 6 months: 3.8 ± 0.8, p=0.01<br><br>Identifying weight-gain-promoting medications (mean ± SD)<br>Control group<br>• Baseline: 2.9 ± 0.8; 6 months: 3.3 ± 0.8, p=0.13<br>Intervention group<br>• Baseline: 2.7 ± 0.2; 6 months: 3.2 ± 0.8, p=0.02<br><br>Discussing anti-obesity medications (mean ± SD)<br>Control group	

				• Baseline: 2.2 ± 0.9; 6 months: 2.7 ± 1.1, p=0.08<br>Intervention group<br>• Baseline: 1.6 ± 0.1; 6 months: 2.5 ± 0.7, p<0.01<br><br>Discussing bariatric surgery (mean ± SD)<br>Control group<br>• Baseline: 2.8 ± 0.7; 6 months: 3.3 ± 0.8, p=0.06<br>Intervention group<br>• Baseline: 2.5 ± 0.2; 6 months: 3.1 ± 1.1, p<0.01<br><br>Post-op management (mean ± SD)<br>Control group<br>• Baseline: 2.2 ± 0.7; 6 months: 2.8 ± 0.9, p=0.01<br>Intervention group<br>• Baseline: 2.2 ± 0.2; 6 months: 2.5 ± 0.9, p=0.08	
**Authors:**<br>Distor-Castro et al.[25]<br><br>**Year published:**<br>2024<br><br>**Study design:**<br>Before-and-after study and non-randomised controlled trial<br><br>**Country:**<br>United States	**Sample size:**<br>N=101<br><br>**Inclusion criteria:**<br>• Primary care staff (nurses and medical assistants) from the intervention and control clinic<br><br>**Exclusion criteria:**<br>• Not stated<br><br>**Setting and population:**<br>Primary care staff from the intervention clinic and an Internal Medicine clinic.	**Intervention**<br>**Format and mode of delivery:**<br>• In-person PowerPoint presentation<br><br>**Topics covered:**<br>• Obesity<br>• Impact of weight stigma<br>• Weight assessment workflow<br>• Available weight management programmes<br><br>**Duration and frequency:**<br>• One 50-minute presentation<br><br>**Control**<br>No intervention.	**1. Attitudes**<br>• Empathy (how well primary care staff recognise and empathise with the negative experiences of patients with obesity)<br>• Critical health (agreement with the critiques concerning health and obesity)<br>• General attribution complexity (beliefs about the causes of obesity, including whether it is within an individual's control or not)<br><br>Attitudes were measured using 3 subscales in the Fat Attitude Assessment Toolkit (FAAT), which is a validated Likert scale survey (higher score = more positive view on obesity).<br><br>**2. Number of referrals**<br>• Number of patient referrals to weight management programmes<br><br>Number of referrals was measured by collecting referral data from the two participating clinics.	**Data analysis**<br>"A one-sided paired t-test was used to analyse the means of pre- and post-intervention FAAT scores. For the second outcome, patient referrals from physicians from both clinics were collected, and ratios and chi-square analysis were calculated."<br><br>**1. Attitudes**<br>Empathy category (mean ± SD, n = 30)<br>• Baseline: 5.592 ± 1.059<br>• 12 weeks: 5.980 ± 0.6259, p=0.038<br><br>Critical health category (mean ± SD, n = 30)<br>• Baseline: 5.286 ± 1.3211<br>• 12 weeks: 5.747 ± 0.7999, p=0.046<br><br>General attribution complexity (mean ± SD, n = 30)<br>• Baseline: 5.725 ± 0.9241<br>• 12 weeks: 5.933 ± 0.6746, p=0.150<br><br>**2. Number of referrals**<br>Number of weight management programme referrals per physician<br>• Control clinic (n=21): 10.29<br>• Intervention clinic (n=40): 17.85, p<0.05	**Author's conclusions:**<br>"Primary care clinic visits are opportunities to address obesity and its related health risks and appropriate interventions. This project's outcomes highlighted the need to educate and involve the primary care staff in mitigating obesity stigma to help utilize the available weight management programs within the health maintenance organisation. Future projects should explore patient-centred outcomes of weight management programs referrals in relation to obesity sensitivity training."<br><br>**Reviewer's comments:**<br>This study found that a 50-minute PowerPoint

					presentation significantly improved primary care staff's empathy towards patients with obesity and their agreement with critical health perspectives. Notably, the intervention clinic demonstrated a significantly higher number of patient referrals to weight management programmes, indicating a positive impact on clinical practice.<br><br>This study has clearly met 7/9 (78%) criteria in the critical appraisal tool.
**Authors:**<br>Jain et al.[43]<br><br>**Year published:**<br>2020<br><br>**Study design:**<br>Before-and-after study<br><br>**Country:**<br>United States	**Sample size:**<br>N=54<br><br>**Inclusion criteria:**<br>• Internal medicine residents caring for patients at two university clinic sites<br><br>**Exclusion criteria:**<br>• Not stated<br><br>**Setting and population:**<br>Internal medicine residents at two university clinics, Pittsburgh.	**Intervention**<br>**Format and mode of delivery:**<br>• Case-based learning activities delivered during two preclinic conference sessions<br><br>**Topics covered:**<br>• Nonpharmacologic management of patients with hypertension, hyperlipidaemia, and BMI, including dietary patterns<br>• Nutrition counselling based on stage of behaviour change<br>• Current diet trends<br><br>**Duration and frequency:**<br>• Two 45-minute sessions<br><br>**Control**<br>Not applicable.	**1. Knowledge**<br>• Knowledge of nutrition counselling<br><br>Knowledge was assessed by six multiple-choice questions.<br><br>**2. Skills related to nutrition counselling**<br>• Frequency of performing tasks related to nutrition counselling for patients with obesity<br><br>Skills related to nutrition counselling was measured through a self-report survey with the options always, most of the time, never, and some of the time.<br><br>**3. Frequency of documentation**<br>• Obesity or overweight in the problem list or encounter diagnosis<br>• Waist circumference	**Data analysis**<br>Descriptive statistics and the number and frequency of responses on pre-versus post-surveys were compared using McNemar's test.<br><br>**1. Knowledge**<br>Knowledge score (mean)<br>• Baseline (n=54): 67%<br>• Post-intervention (n=24): 85%, p=0.004<br>• 2 months (n=11): 74%<br><br>**2. Skills related to nutrition counselling**<br>Frequency of reporting tasks (n=12, number and percentage of residents responding always or most of the time)<br>Listing weight as a separate problem or diagnosis in the EHR<br>• Baseline: 6/12 (50%); 2 months: 6/12 (50%)<br><br>Measuring waist circumference for additional risk stratification<br>• Baseline: 0/12 (0%); 2 months: 0/12 (0%)	**Author's conclusions:**<br>"We demonstrated improved knowledge of nutrition interventions to reduce cardiovascular risk and reported improvement of resident-provided nutrition counselling for appropriate patients. This activity offers IM residents effective initial nutrition training for patients at risk for cardiovascular disease and is practical to implement as part of an ambulatory curriculum."<br><br>**Reviewer's comments:**<br>This study found that two brief case-based learning sessions significantly

			• Goals made or altered during the visit<br>• Referrals placed for nutrition-related counselling<br>• Provision of nutrition counselling<br><br>Frequency of documentation as measured by reviewing EHRs of patients (n=473). Documentation of obesity/overweight was measured via the ICD-10 code for obesity/overweight.	Taking a diet history when addressing obesity<br>• Baseline: 7/12 (58%); 2 months: 9/12 (75%)<br><br>Using a dietary assessment tool when taking a dietary history<br>• Baseline: 7/12 (58%); 2 months: 9/12 (75%)<br><br>Providing nutrition counselling to patients<br>• Baseline: 7/12 (58%); 2 months: 11/12 (91%)<br><br>Referring appropriate patients for nutrition counselling<br>• Baseline: 5/12 (42%); 2 months: 7/12 (58%)<br><br>**3. Frequency of documentation**<br>Obesity or overweight in the problem list or encounter diagnosis (% EHRs with documentation)<br>Baseline: 35%; 2 months: 37%, p=0.554<br><br>Referrals placed for nutrition counselling (% EHRs with documentation)<br>Baseline: 8%; 2 months: 8%, p=0.968<br><br>Provision of nutrition counselling (% EHRs with documentation)<br>Baseline: 35%; 2 months: 41%, p=0.07	improved healthcare professionals' knowledge of nutrition counselling. While self-reported nutrition counselling skills increased, observed documentation of obesity-related care in electronic health records, including problem listing and referrals, showed no significant improvement.<br><br>This study has clearly met 5/7 (71%) criteria in the critical appraisal tool.
**Authors:**<br>Koran-Scholl et al.[44]<br><br>**Year published:**<br>2023<br><br>**Study design:**<br>Before-and-after study<br><br>**Country:**<br>United States	**Sample size:**<br>N=83<br><br>**Inclusion criteria:**<br>• Family medicine residents from three family medicine residency programmes<br><br>**Exclusion criteria:**<br>• Not stated<br><br>**Setting and population:**	**Intervention**<br>**Format and mode of delivery:**<br>• Web-based module containing five clinical vignettes as part of an in-person didactic<br><br>**Topics covered:**<br>• Biopsychosocial management of obesity<br>• Biological complexity of obesity<br>• Implicit and explicit aspects of bias and mitigation strategies<br><br>**Duration and frequency:**<br>• 15-minute module as part of a 1-hour didactic	**1. Comfort**<br>• Level of comfort working with patients with obesity<br><br>**2. Bias**<br>• Understanding of own biases in working with patients with obesity<br><br>Comfort and bias were measured using a 4-point scale (1 = not at all, 4 = mostly).	**Data analysis**<br>Descriptive statistics. Paired t-tests assessed changes in comfort and understanding of biases from pre- to post-intervention.<br><br>**1. Comfort**<br>Level of comfort working with patients (mean ± SD)<br>• Baseline: 3.00 ± 0.7<br>• Post-intervention:3.30 ± 0.6, p=0.001<br><br>**2. Bias**<br>Understanding of own biases (mean ± SD)<br>• Baseline: 2.77 ± 0.7<br>• Post-intervention: 3.48 ± 0.5, p<0.001	**Author's conclusions:**<br>"This teaching e-module is a short, interactive, web-based educational intervention that is free and opensourced. The first-person patient perspective allows learners to better understand the patient's point of view and its PCMH setting illustrates interactions with a variety of healthcare professionals. It was engaging and well

	Family medicine residents from three family medicine residency programmes, Connecticut.	**Control**<br>Not applicable.			received by family medicine residents. This module can begin the conversation around obesity bias, leading to improved patient care."<br><br>**Reviewer's comments:**<br>This study found that a brief web-based module within a didactic session significantly enhanced healthcare professionals' comfort in working with patients with obesity. It also significantly improved their understanding of their own biases regarding these patients, indicating a positive shift in self-awareness.<br><br>This study has clearly met 5/7 (71%) criteria in the critical appraisal tool.
**Authors:**<br>Neuman et al.[23]<br><br>**Year published:**<br>2024<br><br>**Study design:**<br>Before-and-after study<br><br>**Country:**<br>United States	**Sample size:**<br>N=26<br><br>**Inclusion criteria:**<br>• Second-year residents in an internal medicine programme<br><br>**Exclusion criteria:**<br>• Not stated<br><br>**Setting and population:**<br>Internal medicine residents.	**Intervention**<br>**Format and mode of delivery:**<br>• 10 clinical sessions including educational group discussion, interactive case-based learning, and resident-led, attending-supervised patient visits<br><br>**Topics covered:**<br>• Weight loss therapies and management of common weight-related comorbidities (including diet, physical activity, pharmacotherapy, and bariatric surgery)	**1. Competence**<br>• Frequency of discussing how a patient's weight may be affecting their health<br>• Comfort in talking to patients about their weight<br>• Comfort in prescribing medications for the management of obesity<br>• Comfort in prescribing physical activity recommendations for patients with obesity<br>• Comfort in using behavioural interventions to help patients improve food habits<br>• Success in helping patients with obesity lose weight	**Data analysis**<br>"Mean differences in survey measures at 12 months were analyzed using paired t tests."<br><br>**1. Competence**<br>Discussing how a patient's weight may be affecting their health (mean)<br>• Baseline (n=26): 3.3<br>• Post-intervention (n=14): 4.0<br>• Post-pre mean (95% CI): 0.7 (0.2-1.2)<br><br>Talking to patients about their weight (mean)<br>• Baseline (n=26): 3.3<br>• Post-intervention (n=14): 4.4<br>• Post-pre mean (95% CI): 1.1 (0.8-1.5)	**Author's conclusions:**<br>"The training experience was feasible and acceptable, and demonstrated improvements in resident outcomes and patient weight."<br><br>**Reviewer's comments:**<br>This study found that a year-long clinical training programme significantly improved healthcare professionals' competence across various aspects of

		• Overview of obesity and patient evaluation<br>• Disordered eating, metabolic syndrome, obstructive sleep apnoea, endocrine disorders<br><br>**Duration and frequency:**<br>• 10 half-day sessions delivered every 5 weeks over 12 months<br>• Each session included 30-minutes of educational group discussion with case-based learning and 4 hours of patient visits<br><br>**Control**<br>Not applicable.	Competence was measured using a 5-point survey (1 = rarely/very uncomfortable, 5 = consistently/ very comfortable).<br><br>**2. Knowledge**<br>• Understanding of the effects of obesity on co-morbid conditions<br>• Knowledge of pharmacotherapy options for management of obesity<br>• Understanding of weight loss nutrition<br>• Knowledge of criteria for referring patients for bariatric surgery<br>• Understanding of the mechanisms and biology of obesity and weight homeostasis<br><br>Knowledge was measured using a 5-point survey (1 = poor understanding, 5 = advanced understanding).<br><br>**3. Attitudes**<br>Attitudes towards obesity and treatment<br>• Bias<br>• Biopsychosocial attitudes<br><br>Attitudes were measured using a 5-point survey (1 = not at all/strongly disagree, 5 = extremely/strongly agree).<br><br>**4. Patient outcomes**<br>• Weight loss<br><br>Patient outcomes were measured by extracting patient anthropometric measures from clinic visits.	Prescribing medications for the management of obesity (mean)<br>• Baseline (n=26): 2.2<br>• Post-intervention (n=14): 3.6<br>• Post-pre mean (95% CI): 1.4 (1.1-1.6)<br><br>Prescribing physical activity recommendations for patients with obesity (mean)<br>• Baseline (n=26): 3.3<br>• Post-intervention (n=14): 3.9<br>• Post-pre mean (95% CI): 0.6 (0.2-1.1)<br><br>Using behavioural interventions to help patients improve food habits (mean)<br>• Baseline (n=26): 2.7<br>• Post-intervention (n=14): 3.6<br>• Post-pre mean (95% CI): 1.0 (0.3-1.5)<br><br>Helping patients with obesity lose weight (mean)<br>• Baseline (n=26): 2.7<br>• Post-intervention (n=14): 3.5<br>• Post-pre mean (95% CI): 0.8 (0.4-1.3)<br><br>**2. Knowledge**<br>Understanding of the effects of obesity on co-morbid conditions (mean)<br>• Baseline (n=26): 2.9<br>• Post-intervention (n=14): 3.8<br>• Post-pre mean (95% CI): 0.9 (0.5-1.2)<br><br>Knowledge of pharmacotherapy options for management of obesity (mean)<br>• Baseline (n=26): 2.1<br>• Post-intervention (n=14): 3.6<br>• Post-pre mean (95% CI): 1.5 (1.1-1.9)<br><br>Understanding of weight loss nutrition (mean)<br>• Baseline (n=26): 2.6<br>• Post-intervention (n=14): 3.5<br>• Post-pre mean (95% CI): 0.8 (0.2-1.3)	obesity management, including discussing weight, prescribing medications, and using behavioural interventions. Participants also gained substantial knowledge in pharmacotherapy, bariatric surgery, and the biology of obesity. It also resulted in an average patient weight loss of 4.5kg.<br><br>This study has clearly met 5/7 (71%) criteria in the critical appraisal tool.

				Knowledge of criteria for referring patients for bariatric surgery (mean)<br>• Baseline (n=26): 2.5<br>• Post-intervention (n=14): 3.3<br>• Post-pre mean (95% CI): 0.9 (0.2-1.6)<br><br>Understanding of the mechanisms and biology of obesity and weight homeostasis (mean)<br>• Baseline (n=26): 2.3<br>• Post-intervention (n=14): 3.4<br>• Post-pre mean (95% CI): 0.9 (0.3-1.4)<br><br>**3. Attitudes**<br>"Largely stable over the 12-month program."<br><br>**4. Patient outcomes**<br>Weight loss (mean, 95% CI): 4.5kg (3.0-6.0)	
**Authors:**<br>Phillips et al.[31]<br><br>**Year published:**<br>2022<br><br>**Study design:**<br>Before-and-after study<br><br>**Country:**<br>United States	**Sample size:**<br>N=46[b]<br><br>**Inclusion criteria:**<br>• English proficiency<br>• Qualification as an occupational therapy practitioner<br>• Internet connectivity<br><br>**Exclusion criteria:**<br>• Other healthcare professionals<br>• No access to internet<br><br>**Setting and population:**<br>Occupational therapy practitioners, Boston.	**Intervention**<br>**Format and mode of delivery:**<br>• Seven self-directed modules delivered via an online learning platform (including case-studies, video clips, photos, diagrams, drawn illustrations, statistics, sensitivity strategies, short quizzes)<br><br>**Topics covered:**<br>• General knowledge<br>• Stigma<br>• Occupational therapy and obesity<br>• Safe patient handling<br>• Bariatric equipment<br>• Advocacy<br>• Telehealth<br><br>**Duration and frequency:**<br>• Participants took approximately 1 hour for participants to complete each module (7 hours total), with all modules to be completed within 3 months	**1. Competence**<br>• Evaluating and treating a client with obesity<br>• Having the necessary knowledge base on psychosocial comorbidities related to obesity<br>• Operating and working with bariatric equipment for clients with obesity<br><br>**2. Role of occupational therapy**<br>• Role of occupational therapy practitioners in obesity and bariatric care<br><br>**3. Stigma**<br>• Negative effect of obesity-related stigma with best practice in healthcare settings<br><br>Competence, the role of occupational therapy, and stigma was measured using a 5-point Likert scale (1 = strong disagreement, 5 = strong agreement).	**Data analysis**<br>Paired t-tests compared pre- and post-programme ratings on five Likert-style questions to assess within-group changes.<br><br>**1. Competence**<br>I feel competent enough to evaluate and treat a client with obesity (mean ± SD)<br>• Baseline: 2.9 ± 1.04<br>• Post-intervention: 4.13 ± 0.625, p=0.00001<br><br>I feel I have the necessary knowledge base on psychosocial comorbidities related to obesity (mean ± SD)<br>• Baseline: 2.73 ± 1.09<br>• Post-intervention: 4.22 ± 0.59, p=0.00001<br><br>I feel competent enough to operate and work with bariatric equipment for clients with obesity (mean ± SD)<br>• Baseline: 2.79 ± 1.11<br>• Post-intervention: 3.8 ± 0.91, p=0.00001<br><br>**2. Role of occupational therapy**	**Author's conclusions:**<br>"More education is needed on obesity and bariatric care for occupational therapy practitioners and occupational therapy students. An online education program was demonstrated to be an effective way of increasing knowledge on this important topic to reduce work-related injury."<br><br>**Reviewer's comments:**<br>This study found that a seven-module online programme significantly improved occupational therapists' competence in evaluating and treating clients with obesity, including their knowledge

		**Control**<br>Not applicable.		I feel that occupational therapy practitioners have a positive role in obesity and bariatric care (mean ± SD)<br>• Baseline: 4.25 ± 0.81<br>• Post-intervention: 4.67 ± 0.52, p=0.00175<br><br>**3. Stigma**<br>I feel that obesity related stigma negatively affects best practice in healthcare settings (mean ± SD)<br>• Baseline: 4.13 ± 0.73<br>• Post-intervention: 4.57 ± 0.62, p=0.00248	of psychosocial comorbidities and bariatric equipment. The intervention also improved the perceived positive role of occupational therapy in obesity care and increased awareness of the negative impact of obesity-related stigma in healthcare.<br><br>This study has clearly met 5/7 (71%) criteria in the critical appraisal tool.
**Authors:**<br>Strauss et al.[21]<br><br>**Year published:**<br>2022<br><br>**Study design:**<br>Before-and-after study<br><br>**Country:**<br>United States.	**Sample size:**<br>N=7<br><br>**Inclusion criteria:**<br>• Licensed healthcare professionals<br><br>**Exclusion criteria:**<br>• Not stated<br><br>**Setting and population:**<br>Licensed healthcare professionals.	**Intervention**<br>**Format and mode of delivery:**<br>• Self-paced, online modules, including videos<br><br>**Topics covered:**<br>• Complexities of obesity<br>• Weight bias and its consequences<br><br>**Duration and frequency:**<br>• 5-hour course, to be completed within 4-weeks<br><br>**Control**<br>Not applicable.	**1. Weight bias**<br>• Attitudes towards people with obesity<br>• Belief about the cause of obesity<br><br>Weight bias was measured using the Attitudes Toward Obese Persons (ATOP, higher scores = more positive attitude) and Beliefs About Obese Persons (BAOP, higher score = belief that obesity is not in the person's control) surveys.	**Data analysis**<br>The Wilcoxon signed-rank test compared ATOP and BAOP scores before and after the intervention.<br><br>**1. Weight bias**<br>Attitudes towards people with obesity (median)<br>• Baseline: 14<br>• Post-intervention: 22, p<0.05<br><br>Belief about the cause of obesity (median)<br>• Baseline: -5<br>• Post-intervention: 3, p<0.05	**Author's conclusions:**<br>"This project demonstrated that obesity and weight bias modules can reduce weight bias in HCPs. Future research and quality improvement projects are needed to identify the impact of online training on quality of care and care seeking behaviours of patients with obesity."<br><br>**Reviewer's comments:**<br>This study found that a five-hour self-paced online course significantly mitigated healthcare professionals' weight bias. Post-intervention, participants exhibited more positive attitudes towards people with obesity and a stronger belief that obesity is not

					solely within an individual's control.<br><br>This study has clearly met 5/7 (71%) criteria in the critical appraisal tool.
**Authors:**<br>Velazquez et al.[46]<br><br>**Year published:**<br>2024<br><br>**Study design:**<br>Before-and-after study and non-randomised controlled trial<br><br>**Country:**<br>United States	**Sample size:**<br>N=307<br><br>**Inclusion criteria:**<br>• Healthcare professionals working within a specific medical centre service region<br><br>**Exclusion criteria:**<br>• Healthcare professionals who did not have a scope of practice requiring obesity care (such as inpatient, paediatrics, radiology, administration, and surgery)<br><br>**Setting and population:**<br>Healthcare professionals working within a specific medical centre service region, California.	**Intervention**<br>**Format and mode of delivery:**<br>• Symposium, including patient panel discussions, faculty-led didactics, video viewing, question/answer periods, and simulated clinical scenarios<br><br>**Topics covered:**<br>• Responsibility of obesity<br>• Biological and structural factors that determine weight<br>• Negative treatment experiences of patients from HCPs<br>• Bariatric surgery<br>• Impact of weight bias<br><br>**Duration and frequency:**<br>• One 4-hour symposium<br><br>**Control**<br>No intervention.	**1. Weight bias**<br>• Negative obesity stereotypes<br>• Empathy for patients with obesity<br>• Confidence in clinical interaction with patients with obesity<br><br>Weight bias was measured using a 16-item questionnaire from Kushner and colleagues, which used a 5-point Likert scale (1=strongly disagree, 5=strongly agree).<br><br>**2. Practice behaviours**<br>• Obesity diagnosis rates<br>• Referral rates to a healthy lifestyle programme, obesity medicine, and bariatric surgery<br><br>Practice behaviours were measured by review of electronic medical records.	**Data analysis**<br>Unadjusted differences were assessed using paired and independent samples t-tests. Nonparametric Fisher's exact tests were used for comparisons of categorical data.<br><br>**1. Weight bias**<br>Negative obesity stereotypes (mean ± SD)<br>• Baseline (n=218): 2.81 ± 0.47<br>• Post-intervention (n=183): 2.50 ± 0.46, p<0.001<br>• 4-months (n=79): 2.53 ± 0.48, p=0.75<br>• 12-months (n=44): 2.93 ± 1.35, p=0.08<br><br>Empathy (mean ± SD)<br>• Baseline (n=218): 3.32 ± 0.64<br>• Post-intervention (n=183): 3.47 ± 0.63, p=0.006<br>• 4-months (n=79): 3.45 ± 0.68, p=0.06<br>• 12-months (n=44): 3.48 ± 0.59, p=0.39<br><br>Confidence in treating (mean ± SD)<br>• Baseline (n=218): 3.10 ± 0.86<br>• Post-intervention (n=183): 3.85 ± 0.79, p<0.001<br>• 4-months (n=79): 3.81 ± 0.79, p=0.66<br>• 12-months (n=44): 3.75 ± 0.90, p=0.60<br><br>Overall score (mean ± SD)<br>• Baseline (n=218): 2.97 ± 0.35<br>• Post-intervention (n=183): 2.93 ± 0.33, p=0.45<br>• 4-months (n=79): 2.94 ± 0.37, p=0.85<br>• 12-months (n=44): 2.96 ± 0.38, p=0.76<br><br>**2. Practice behaviours**<br>Diagnosing patients with obesity (% of patients the behaviour was performed on)<br>Control group (n=89)<br>• Baseline: 21.1%	**Author's conclusions:**<br>"This intervention has promise to be a scalable program that goes beyond impacting HCP's self-reported weight bias and also changes HCPs' clinical practice behaviours related to obesity treatment."<br><br>**Reviewer's comments:**<br>This study found that a four-hour symposium led to a significant increase in obesity diagnosis rates and referrals to healthy lifestyle programmes, obesity medicine clinics, and bariatric surgery. While it reduced HCPs' negative stereotypes about obesity and improved their confidence in clinical interactions immediately post-intervention, it did not lead to a significant reduction in overall weight bias.<br><br>This study has clearly met 8/9 (89%) criteria in the critical appraisal tool.

				• 12-months: 17.8%, p<0.001<br>Intervention group (n=218)<br>• Baseline: 25.9%<br>• 12-months: 27.3%, p<0.001<br>Between-group difference at 12-months (adjusted OR, 95% CI)<br>• Intervention group: 1.60 (1.54, 1.66)<br><br>Referring patients to healthy lifestyle programmes (% of patients the behaviour was performed on)<br>Control group (n=89)<br>• Baseline: 6.2%<br>• 12-months: 5.0%, p<0.001<br>Intervention group (n=218)<br>• Baseline: 5.2%<br>• 12-months: 6.7%, p<0.001<br>Between-group difference at 12-months (adjusted OR, 95% CI)<br>• Intervention group: 1.27 (1.19, 1.36)<br><br>Referring patients to an obesity medicine clinic (% of patients the behaviour was performed on)<br>Control group (n=89)<br>• Baseline: 1.0%<br>• 12-months: 1.0%, p=0.75<br>Intervention group (n=218)<br>• Baseline: 1.5%<br>• 12-months: 2.5%, p<0.001<br>Between-group difference at 12-months (adjusted OR, 95% CI)<br>• Intervention group: 1.87 (1.63, 2.14)<br><br>Referring patients to bariatric surgery (% of patients the behaviour was performed on)<br>Control group (n=89)<br>• Baseline: 1.1%<br>• 12-months: 0.8%, p=0.02<br>Intervention group (n=218)<br>• Baseline: 1.3%<br>• 12-months: 2.1%, p<0.001<br>Between-group difference at 12-months (adjusted OR, 95% CI)	

				• Intervention group: 2.12 (1.70, 2.67)	
**Authors:**<br>Verma et al.[32]<br><br>**Year published:**<br>2021<br><br>**Study design:**<br>Before-and-after study<br><br>**Country:**<br>Malaysia	**Sample size:**<br>N=30<br><br>**Inclusion criteria:**<br>• Community pharmacists currently practicing in Klang Valley, Malaysia<br><br>**Exclusion criteria:**<br>• Not stated<br><br>**Setting and population:**<br>Community pharmacists, Malaysia	**Intervention**<br>**Format and mode of delivery:**<br>• In-person workshop, including didactic lectures, case studies, and small group discussions<br><br>**Topics covered:**<br>• Overview of overweight and obesity<br>• Developing an individualised care plan<br>• Dietary approaches and exercises in weight management<br>• Behavioural modifications in weight management<br>• Pharmacotherapy in weight management<br>• Roles of CPs in the management of overweight and obese patients<br><br>**Duration and frequency:**<br>• One 8-hour event<br><br>**Control**<br>Not applicable.	**1. Knowledge**<br>• Overview of overweight and obesity<br>• Diet, nutrition, and physical activity<br>• Behavioural aspects of obesity and its management,<br>• Pharmacotherapy of overweight and obesity<br><br>Knowledge was measured using a 20-item true/false questionnaire. Each correct response was scored 1, each incorrect response was scored 0.<br><br>**2. Attitudes**<br>• Attitudes towards weight management and weight management training programmes[d]<br><br>Attitudes were measured using a 5-point Likert scale (1=strongly disagree, 5=strongly agree).<br><br>**3. Perceived barriers**<br>• Perceived barriers regarding weight management[e]<br><br>Perceived barriers were measured using a 5-point Likert scale (1=strongly disagree, 5=strongly agree).	**Data analysis**<br>The Wilcoxon signed-rank tests were used to assess within-group differences for outcome variables.<br><br>**1. Knowledge**<br>Overview of overweight and obesity (mean ± SD)<br>• Baseline: 6.40 ± 0.81<br>• Post-intervention: 6.53 ± 0.63, p=0.973<br>• 30 days: 6.80 ± 0.50, p=0.040<br><br>Diet, nutrition, and physical activity (mean ± SD)<br>• Baseline: 3.07 ± 2.16<br>• Post-intervention: 2.76 ± 1.17, p=0.657<br>• 30 days: 3.40 ± 0.91, p=0.018<br><br>Behavioural intervention (mean ± SD)<br>• Baseline: 2.20 ± 0.55<br>• Post-intervention: 2.76 ± 0.86, p=0.003<br>• 30 days: 3.36 ± 0.95, p=0.001<br><br>Pharmacotherapy (mean ± SD)<br>• Baseline: 2.83 ± 0.75<br>• Post-intervention: 2.87 ± 0.68, p=0.843<br>• 30 days: 3.48 ± 0.71, p=0.007<br><br>Overall score (mean ± SD)<br>• Baseline: 14.50 ± 2.45<br>• Post-intervention: 14.93 ± 1.62, p=0.233<br>• 30 days: 17.04 ± 2.51, p=0.001<br><br>**2. Attitudes**<br>I think minor weight loss can produce good clinical outcomes for overweight and obese patients (mean ± SD)<br>• Baseline: 4.07 ± 0.64<br>• Post-intervention: 4.23 ± 0.50, p=0.166<br>• 30 days: 4.52 ± 0.59, p=0.012<br><br>I think I am professionally competent to treat patients with BMI more than 30kg/m^2 (mean ± SD)<br>• Baseline: 3.13 ± 0.97	**Author's conclusions:**<br>"This study showed the potential positive impact of an educational training program on community pharmacists' knowledge, attitudes and perceived barriers towards weight management service."<br><br>**Reviewer's comments:**<br>This study found that an 8-hour workshop significantly improved healthcare professionals' knowledge of behavioural interventions and pharmacotherapy for obesity management at 30 days. Participants' attitudes became more positive regarding the clinical outcomes of minor weight loss and their professional competence in treating obesity. This also led to a significant reduction in the perceived barrier of lack of training.<br><br>This study has clearly met 5/7 (71%) criteria in the critical appraisal tool.

				• Post-intervention: 3.63 ± 0.81, p=0.013<br>• 30 days: 4.04 ± 1.14, p=0.001<br><br>I think adults with BMI above 30 kg/m^2 should be offered anti-obesity drugs (mean ± SD)<br>• Baseline: 3.47 ± 0.94<br>• Post-intervention: 3.97 ± 0.89, p=0.007<br>• 30 days: 4.48 ± 0.82, p=0.001<br><br>**3. Perceived barriers**<br>Lack of training sessions in the management of overweight/obesity (mean ± SD)<br>• Baseline: 3.30 ± 1.02<br>• Post-intervention: 2.93 ± 1.17, p=0.073<br>• 30 days: 2.33 ± 0.70, p=0.001<br><br>Reluctance among overweight/obese patients to pay pharmacists for weight management services (mean ± SD)<br>• Baseline: 2.67 ± 0.99<br>• Post-intervention: 2.93 ± 0.98, p=0.340<br>• 30 days: 2.21 ± 0.42, p=0.030<br><br>Preference among overweight/obese patients to purchase anti-obesity medications instead of getting advice on lifestyle/dietary modification (mean ± SD)<br>• Baseline: 3.23 ± 1.04<br>• Post-intervention: 3.33 ± 0.92, p=0.593<br>• 30 days: 2.33 ± 0.57, p=0.001	
**Authors:**<br>Wharton et al.[45]<br><br>**Year published:**<br>2022<br><br>**Study design:**<br>Before-and-after study<br><br>**Country:**<br>Canada	**Sample size:**<br>N=91<br><br>**Inclusion criteria:**<br>• Canadian family physicians, based on their interest in obesity management and participation in previous obesity management-related programmes and initiatives	**Intervention**<br>**Format and mode of delivery:**<br>• Online, self-directed learning platform, individualised to the physician, including didactic videos and focus group meetings<br><br>**Topics covered:**<br>• Understanding obesity<br>• Communication and behaviour change<br>• Lifestyle interventions<br>• Medical and surgical treatments	**1. Obesity management practices**<br>• Current management practices and changes to intentions in obesity management practices<br><br>Obesity management practices were measured via a self-directed retrospective patient chart review for 10 patients with obesity.	**Data analysis**<br>Descriptive statistics only.<br><br>**1. Obesity management practices**<br>Use of dietary changes<br>Baseline:47%<br>Post-intervention: 29%<br><br>Use of physical activity changes<br>Baseline:37%<br>Post-intervention: 27%<br><br>Pharmacotherapy	**Author's conclusions:**<br>"The program was overall well received and successfully changed obesity management intentions among participating FPs, thus setting the stage for a larger more comprehensive study to examine the efficacy of i-ACT™ in Obesity in addressing knowledge

	**Exclusion criteria:**<br>• Not stated<br><br>**Setting and population:**<br>Family physicians, Canada	• Long-term weight management<br><br>**Duration and frequency:**<br>• Not stated<br><br>**Control**<br>Not applicable.		Baseline:12%<br>Post-intervention: 22%<br><br>Bariatric surgery<br>Baseline:3%<br>Post-intervention: 4%	gaps and advancing evidence-based, guidelines-aligned approach to obesity treatment."<br><br>**Reviewer's comments:**<br>This study found that an online, self-directed learning programme for physicians on obesity management was associated with observed shifts in clinical practice. Post-intervention, a decrease in the documented use of dietary and physical activity interventions was noted, alongside an increase in the use of pharmacotherapy and bariatric surgery.<br><br>This study has clearly met 5/7 (71%) criteria in the critical appraisal tool.
**Authors:**<br>Zevin et al.[26]<br><br>**Year published:**<br>2023<br><br>**Study design:**<br>Before-and-after study and non-randomised controlled trial<br><br>**Country:**<br>Canada	**Sample size:**<br>N=18<br><br>**Inclusion criteria:**<br>• Primary care providers<br><br>**Exclusion criteria:**<br>• Not stated<br><br>**Setting and population:**<br>Primary care providers (including family physicians, nurse	**Intervention**<br>**Format and mode of delivery:**<br>• In-person CPD programme, including didactic teaching, case-based learning, and small group discussions<br><br>**Topics covered:**<br>• Confronting stigma in obesity management<br>• Approach to taking a detailed history<br>• Nutrition and dietary approaches to obesity management<br>• Pharmacotherapy	**1. Knowledge, attitudes, and skills**<br>• Self-reported comfort with learning objectives, confidence, and knowledge in treating patients living with obesity<br><br>Knowledge, attitudes, and skills were measured using a questionnaire with Likert and Likert-like items and narrative comments.<br><br>**2. Behaviour**<br>• Self-reported expected change in behaviours	**Data analysis**<br>Descriptive statistics. "For both methods, measures of central tendency were report, and a paired t test was used to determine if there were any significant differences in referrals to the OBN pre and post CPD intervention and between exposed and unexposed."<br><br>**1. Knowledge, attitudes, and skills**<br>Enhanced knowledge<br>• Agreed/strongly agreed: 15/16 (94%)<br>• Somewhat agreed: 1/16 (6%)<br><br>**2. Behaviour**<br>Intention to make changes to practice<br>• Agreed: 15/16 (94%)	**Author's conclusions:**<br>"The CPD program in our study was overwhelmingly well received and participants reported knowledge (Level 2) and behavioural (Level 3) changes following participation; however, there was no detectable change in their clinical practice (Level 4). The methodology described in our proof-of-concept study can be

	practitioners, nurses, registered dietitians, and social workers), Canada.	• Integration of exercise programmes<br>• Psychological aspects of weight management<br>• Role of bariatric surgery<br><br>**Duration and frequency:**<br>• One 4.5 hour-event<br><br>**Control**<br>No intervention.	Behaviour was measured using a questionnaire with Likert and Likert-like items and narrative comments.<br><br>**3. Clinical practice**<br>• Number of referrals made to the Ontario Bariatric Network for medical and/or surgical weight loss interventions for patients living with severe obesity<br><br>Clinical practice was measured using primary care provider data in the Ontario Bariatric Network referral database, for one year before (pre-intervention) and one year after (post-intervention) the programme.	**3. Change in clinical practice**<br>Number of patients referred by each primary care provider (median, IQR)<br>• Pre-intervention (n=16): 3.5 (1.5)<br>• Post-intervention (n=16): 1.0 (2.0), p=0.32<br><br>• Control group post-intervention (n=18): 3.5 (3.0)<br>• Intervention group post-intervention (n=13): 2.0 (1.0)<br>• Between-group difference: p<0.01	modified and adopted to evaluate Level-4 outcomes in other studies of effectiveness of CPD interventions."<br><br>**Reviewer's comments:**<br>This study found that a single 4.5 hour in-person CPD programme on obesity management reportedly enhanced primary care providers' knowledge, comfort, and intention to change practice, with nearly all agreeing. However, referrals to bariatric surgery significantly decreased post-intervention compared to a control group.<br><br>This study has clearly met 6/9 (67%) criteria in the critical appraisal tool.

Notes:
[a]Includes secretaries and managerial staff
[b]Includes both occupational therapy practitioners and students
[c]The three outcomes most relevant to obesity stigma were extracted
[d]Three questions were extracted outcomes relating to attitudes
[e]Three questions were extracted outcomes relating to perceived barriers

Abbreviations: ASD: autism spectrum disorder; BMI: body mass index; CPD: continuing professional development; EHR: electronic health record; GP: general practitioner; MI: motivational interviewing; OA: osteoarthiritis; OR: odds ratio; RCT: randomised controlled trial; SD: standard deviation

Supplementary material S3: Risk of Bias assessment of RCTs

RCTs		Internal validity bias related to:													
	**Domain**	**Selection and allocation**			**Administration of intervention/exposure**			**Assessment, detection, and measurement of the outcome**			**Participant retention**	**Statistical conclusion validity**			**Overall quality**
	**Question number**	**1**	**2**	**3**	**4[a]**	**5**	**6**	**7**	**8**	**9**	**10**	**11**	**12**	**13**	
**Study**	**Outcome**														
Agung et al. (2025)[24]	Knowledge	Unclear	Unclear	Yes	NA	No	Yes	NA	Yes	NA	No	Yes	Unclear	Yes	7/12
	Clinical skills							Yes	Yes	Yes			Unclear		
Allison et al. (2023)[19]	Confidence	Yes	Unclear	Yes	NA	NA	Yes	NA	Yes	NA	Yes	Yes	Yes	Yes	8/9
	Competence							NA	Yes	NA			Yes		
	Attitudes							NA	Yes	NA			Yes		
Isom et al. (2020)[18]	Obesity knowledge	Yes	Yes	Yes	NA	No	Yes	NA	Yes	NA	No	Yes	Yes	Yes	8/10
	Bias							NA	Yes	NA			Yes		
Sherf-Dagan et al. (2022)[27]	Attitudes	Yes	Unclear	Yes	NA	NA	Yes	NA	Yes	NA	No	Yes	Unclear	Yes	6/9
	Beliefs							NA	Yes	NA			Unclear		

[a]All studies were marked as NA, as blinding participants is not feasible due to the nature of educational interventions

Definitions: NA: not applicable; RCT: randomised controlled trial; JBI critical appraisal tool for the assessment of risk of bias for RCTs

Supplementary material S4: Risk of Bias assessment of quasi-experimental studies

Quasi-experimental studies		Internal validity bias related to:									
	**Domain**	**Temporal precedence**	**Selection and allocation**	**Confounding factors**	**Administration of intervention/exposure**	**Assessment, detection, and measurement of the outcome**			**Participant retention**	**Statistical conclusion validity**	**Overall quality**
	**Question number**	**1**	**2**	**3**	**4**	**5**	**6**	**7**	**8**	**9**	
**Study**	**Outcome**										
Baillargeon et al. (2020)[22]	Attitudes	Yes	No	Yes	NA	Yes	NA	Yes	Unclear	Unclear	4/7
	Confidence					Yes	NA	Yes		Unclear	
	Self-efficacy					Yes	NA	Yes		Unclear	
	Practice changes					Yes	NA	Yes		Unclear	
	Patient outcomes					Yes	NA	Yes		Unclear	
Bernard-Genest et al. (2020)[17]	Self-efficacy	Yes	No	Yes	NA	Yes	NA	Yes	Yes	Unclear	5/7
Bonder et al. (2020)[30]	Self-efficacy	Yes	No	Yes	NA	Yes	NA	Yes	Yes	Unclear	5/7
	Clinical skills					Yes	NA	Yes			
Chae et al. (2024)[20]	Self-efficacy	Yes	Yes	Unclear	Yes	Yes	Yes	Yes	No	Unclear	6/9
	Clinical practice habits					Yes	Yes	Yes		Unclear	
Distor Castro et al. (2024)[25]	Attitudes	Yes	Yes	Unclear	Yes	Yes	Yes	Yes	Yes	Unclear	7/9
	Number of referrals					No	Yes	NA		Unclear	
Jain et al. (020)[43]	Knowledge	Yes	No	Yes	NA	Yes	NA	Yes	Yes	Unclear	5/7
	Skills related to nutrition counselling					Yes	NA	Yes		Unclear	
	Frequency of documentation					Yes	NA	NA		Unclear	
Koran-Scholl et al. (2023)[44]	Comfort	Yes	No	Yes	NA	Yes	NA	Yes	Yes	Unclear	5/7
	Bias					Yes	NA	Yes		Unclear	
Neuman et al. (2024)[23]	Competence	Yes	No	Yes	NA	Yes	NA	Yes	Yes	Unclear	5/7
	Knowledge					Yes	NA	Yes		Unclear	
	Attitudes					Yes	NA	Yes		Unclear	
	Patient outcomes					Yes	NA	Yes		Unclear	
	Competence	Yes	No	Yes	NA	Yes	NA	Yes	Yes	Unclear	5/7

Phillips et al. (2022)[31]	Role of occupational therapy					Yes	NA	Yes		Unclear	
	Stigma					Yes	NA	Yes		Unclear	
Strauss et al. (2022)[21]	Weight bias	Yes	No	Yes	NA	Yes	NA	Yes	Yes	Unclear	5/7
Velazquez et al. (2024)[46]	Weight bias	Yes	Yes	Yes	Yes	Yes	NA	Yes	Yes	Unclear	8/9
	Practice behaviours					Yes	Yes	Yes		Unclear	
Verma et al. (2021)[32]	Knowledge	Yes	No	Yes	NA	Yes	NA	Yes	Yes	Unclear	5/7
	Attitudes					Yes	NA	Yes		Unclear	
	Perceived barriers					Yes	NA	Yes		Unclear	
Wharton et al. (2022)[45]	Obesity management practices	Yes	No	Yes	NA	Yes	NA	Yes	Yes	Unclear	5/7
Zevin et al. (2023)[26]	Knowledge, attitudes, and skills	Yes	Yes	Unclear	Yes	No	NA	Yes	Yes	Unclear	6/9
	Behaviour					No	NA	Yes		Unclear	
	Clinical practice					Yes	Yes	NA		Unclear	

Definitions: NA: not applicable; JBI critical appraisal tool for the assessment of risk of bias for quasi-experimental studies

Supplementary material S5: Subgroup analyses by outcome domain

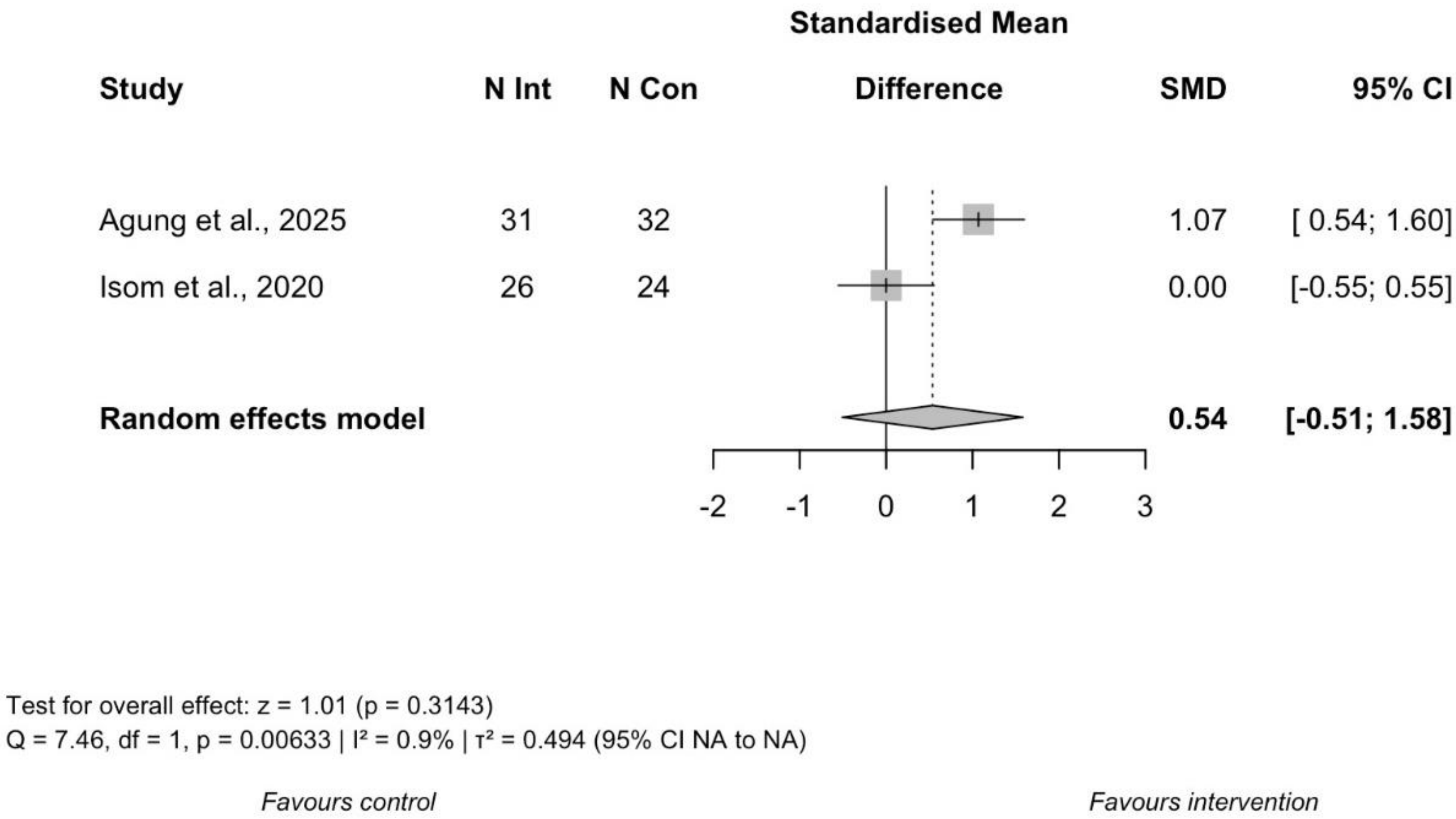

Figure 1. Forest plot of pooled effect sizes for the knowledge domain subgroup

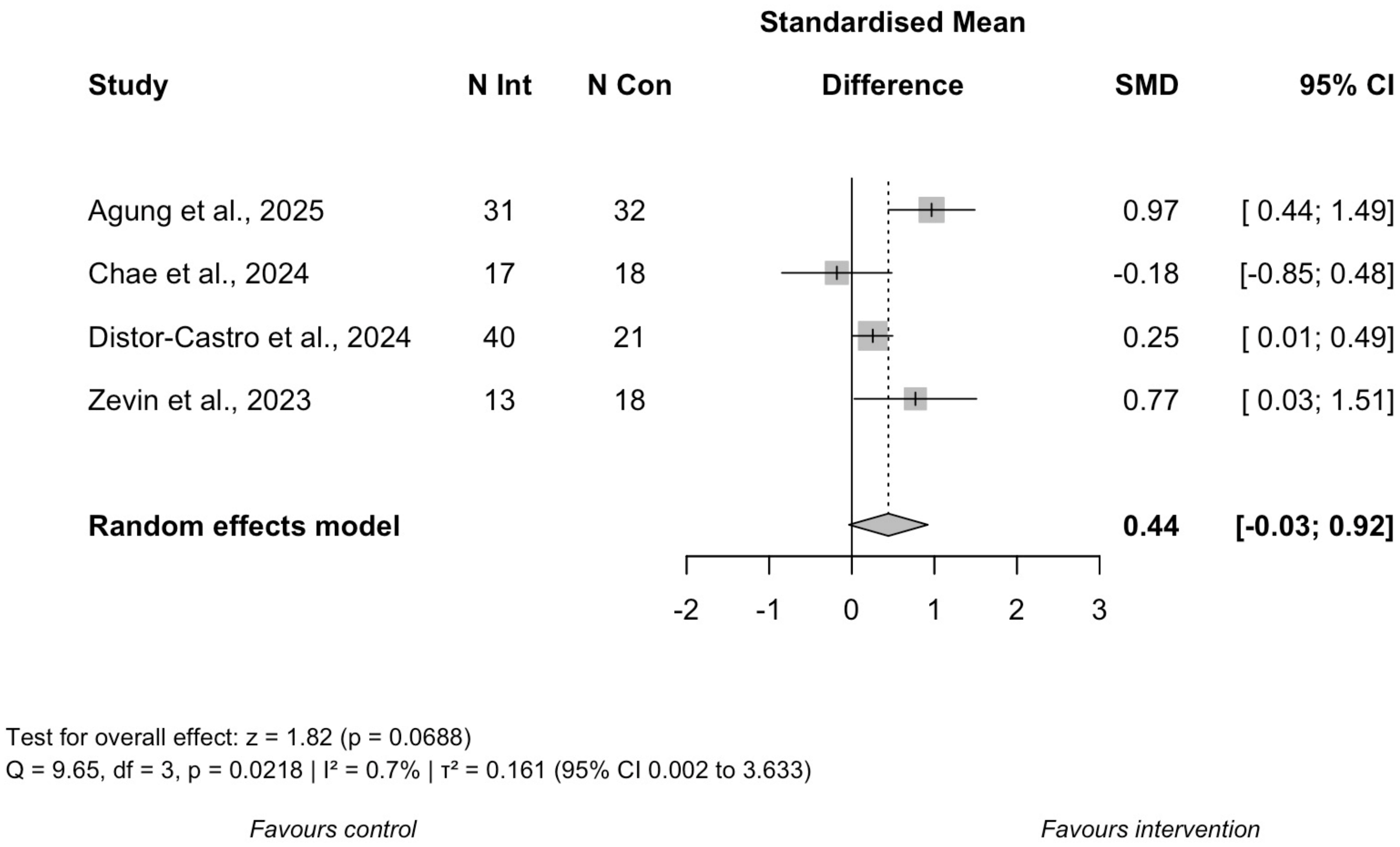

Figure 2. Forest plot of pooled effect sizes for the skills and behaviours domain subgroup

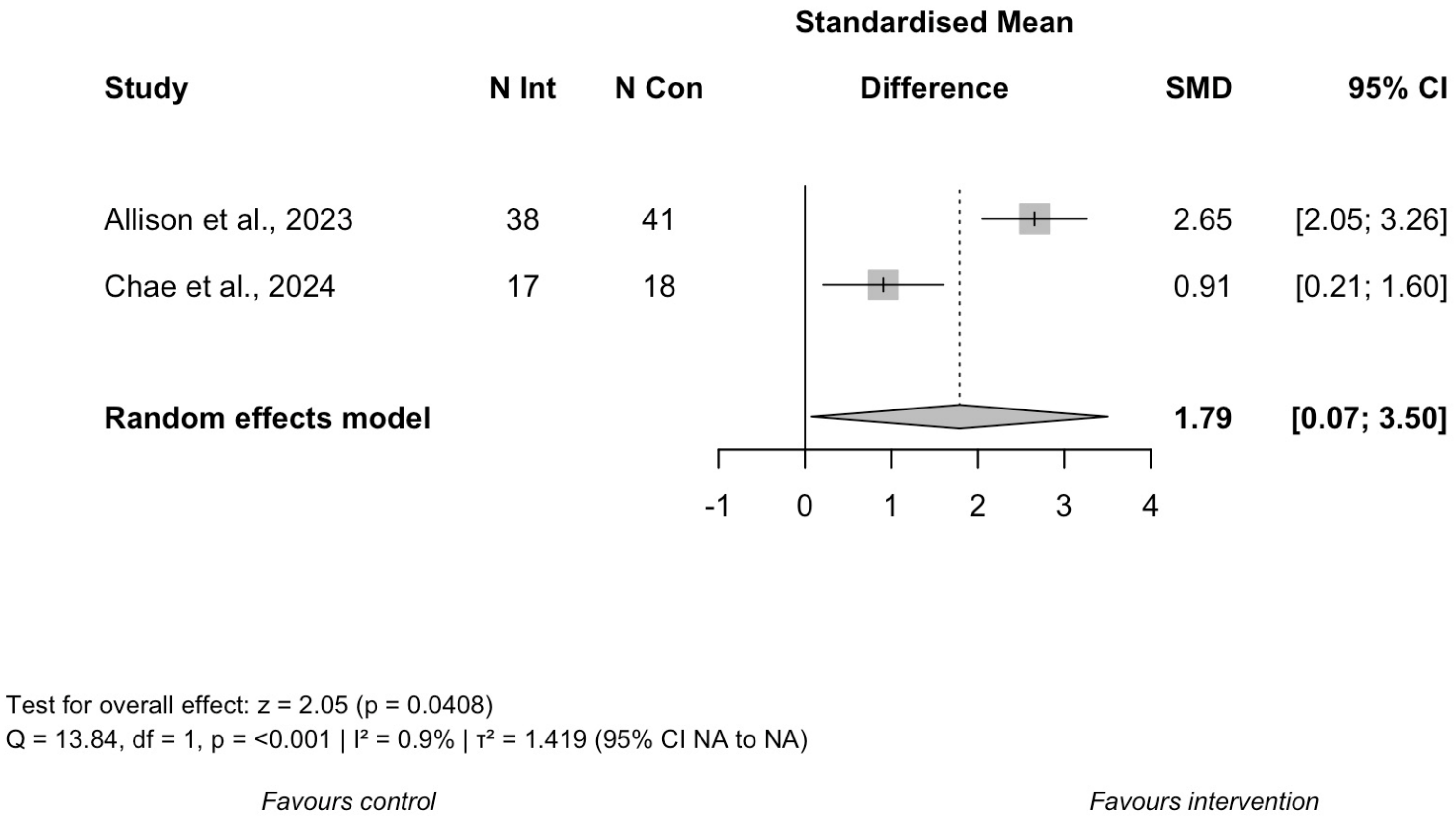

Figure 3. Forest plot of pooled effect sizes for the confidence domain subgroup

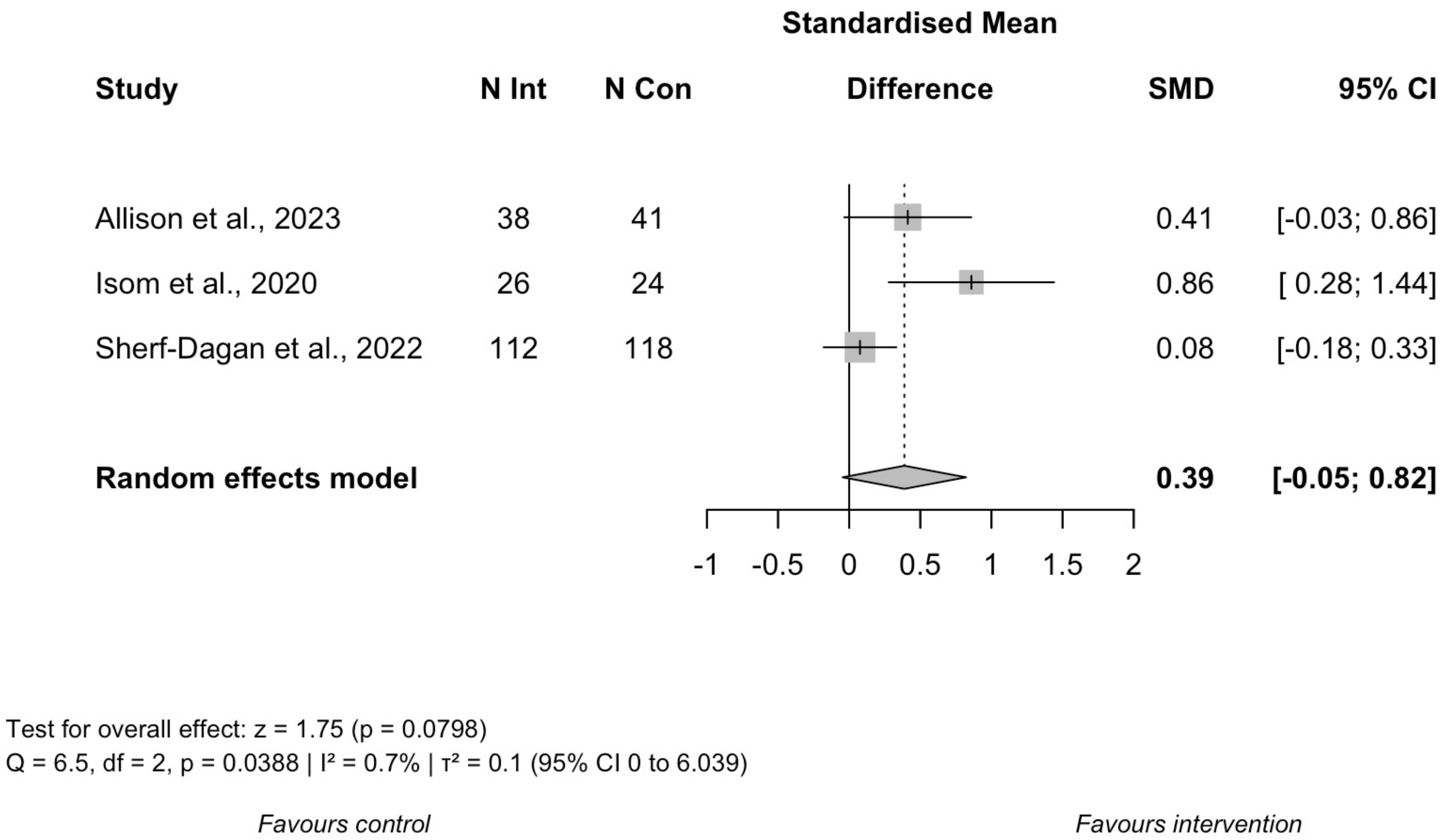

Figure 4. Forest plot of pooled effect sizes for the attitudes domain subgroup

Supplementary material S6: Sensitivity analyses of the primary meta-analysis

(a)

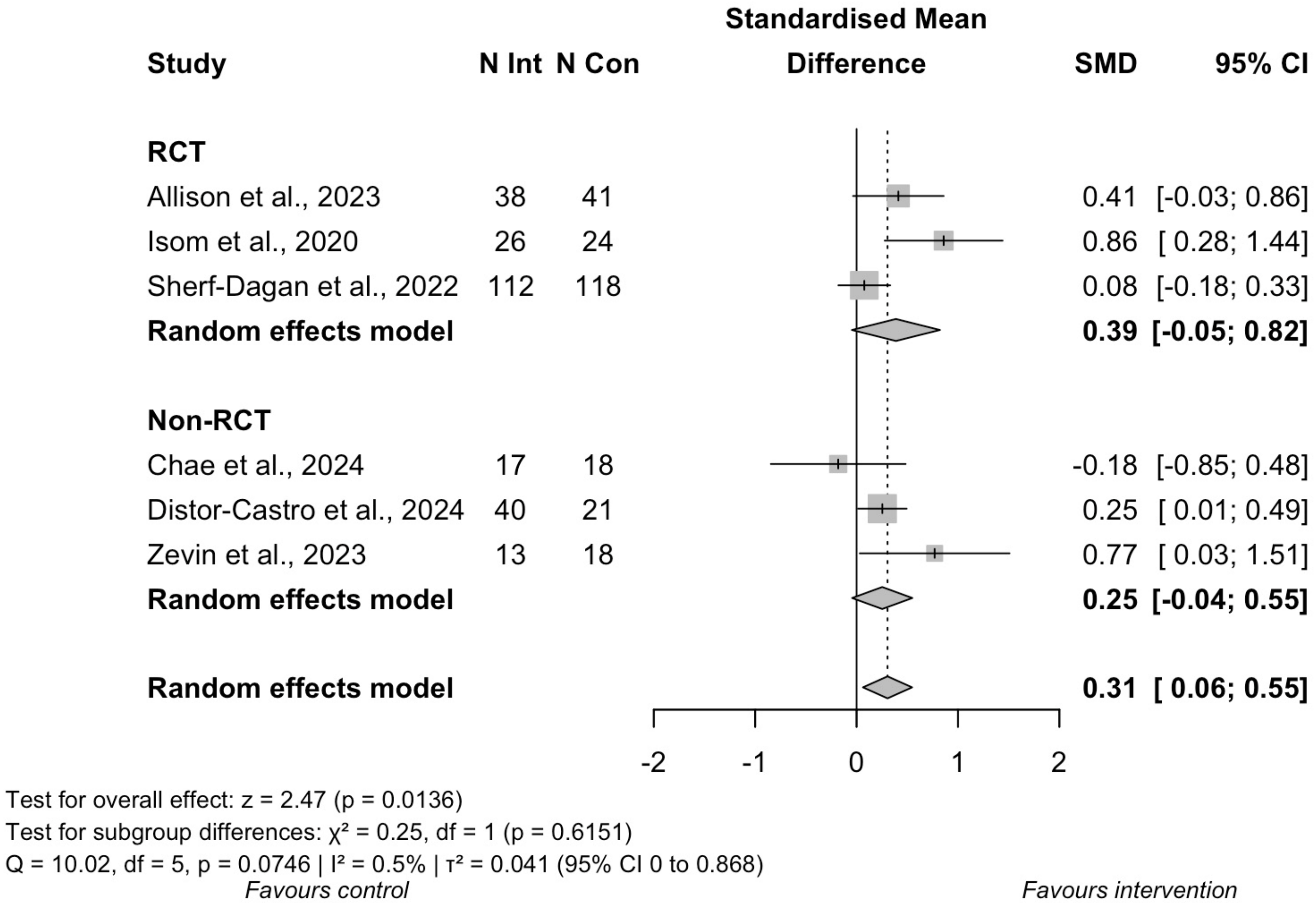

(b)

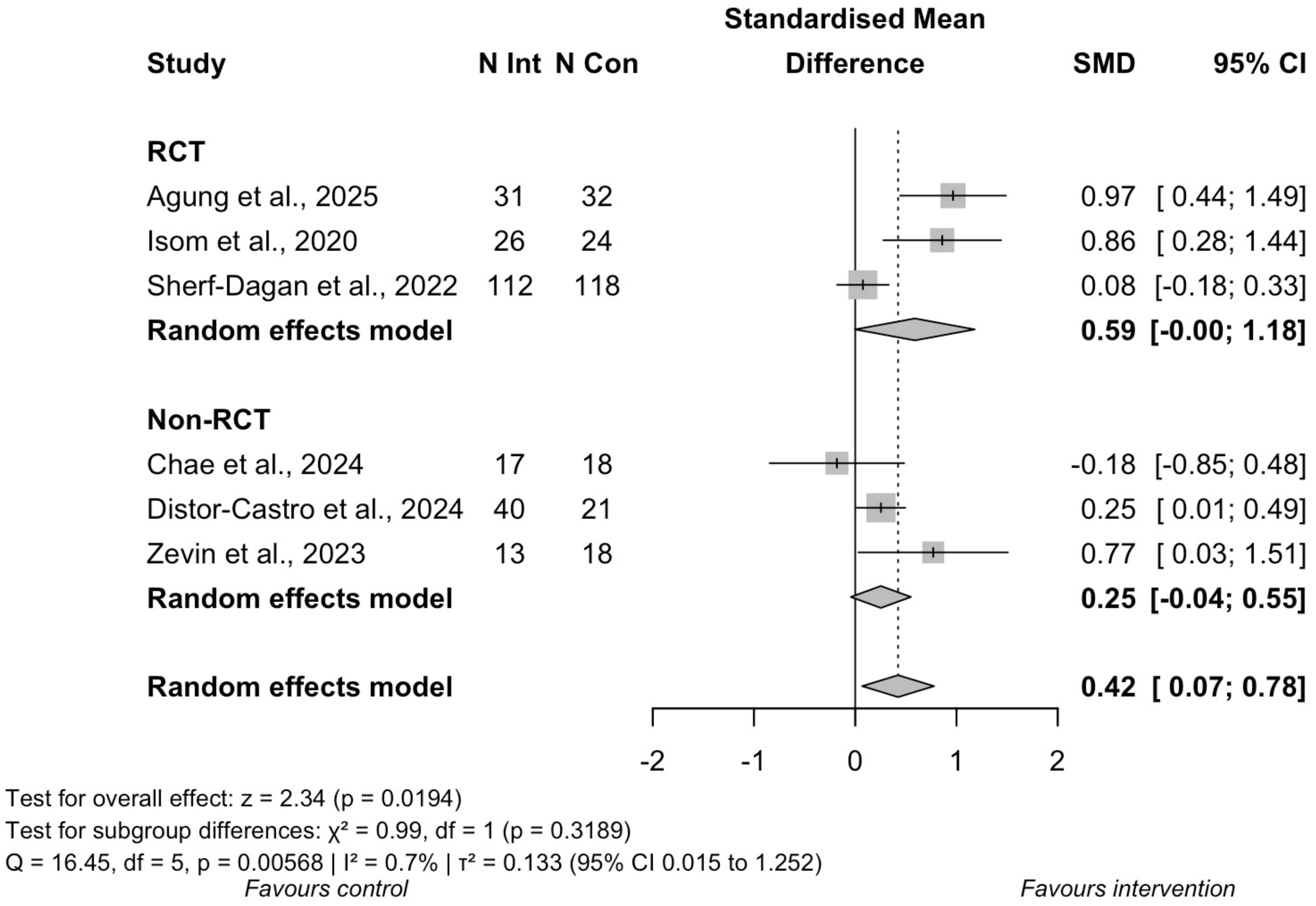
Standardised Mean
Study
N Int
N Con
Difference
SMD
95% CI
RCT
Agung et al., 2025 31 32 0.97 [0.44; 1.49]
Isom et al., 2020 26 24 0.86 [0.28; 1.44]
Sherf-Dagan et al., 2022 112 118 0.08 [-0.18; 0.33]
Random effects model 0.59 [-0.00; 1.18]
Non-RCT
Chae et al., 2024 17 18 -0.18 [-0.85; 0.48]
Distor-Castro et al., 2024 40 21 0.25 [0.01; 0.49]
Zevin et al., 2023 13 18 0.77 [0.03; 1.51]
Random effects model 0.25 [-0.04; 0.55]
Random effects model 0.42 [0.07; 0.78]
-2
-1
0
1
2
Test for overall effect: z = 2.34 (p = 0.0194)
Test for subgroup differences: χ² = 0.99, df = 1 (p = 0.3189)
Q = 16.45, df = 5, p = 0.00568 | I² = 0.7% | τ² = 0.133 (95% CI 0.015 to 1.252)
Favours control
Favours intervention

(c)

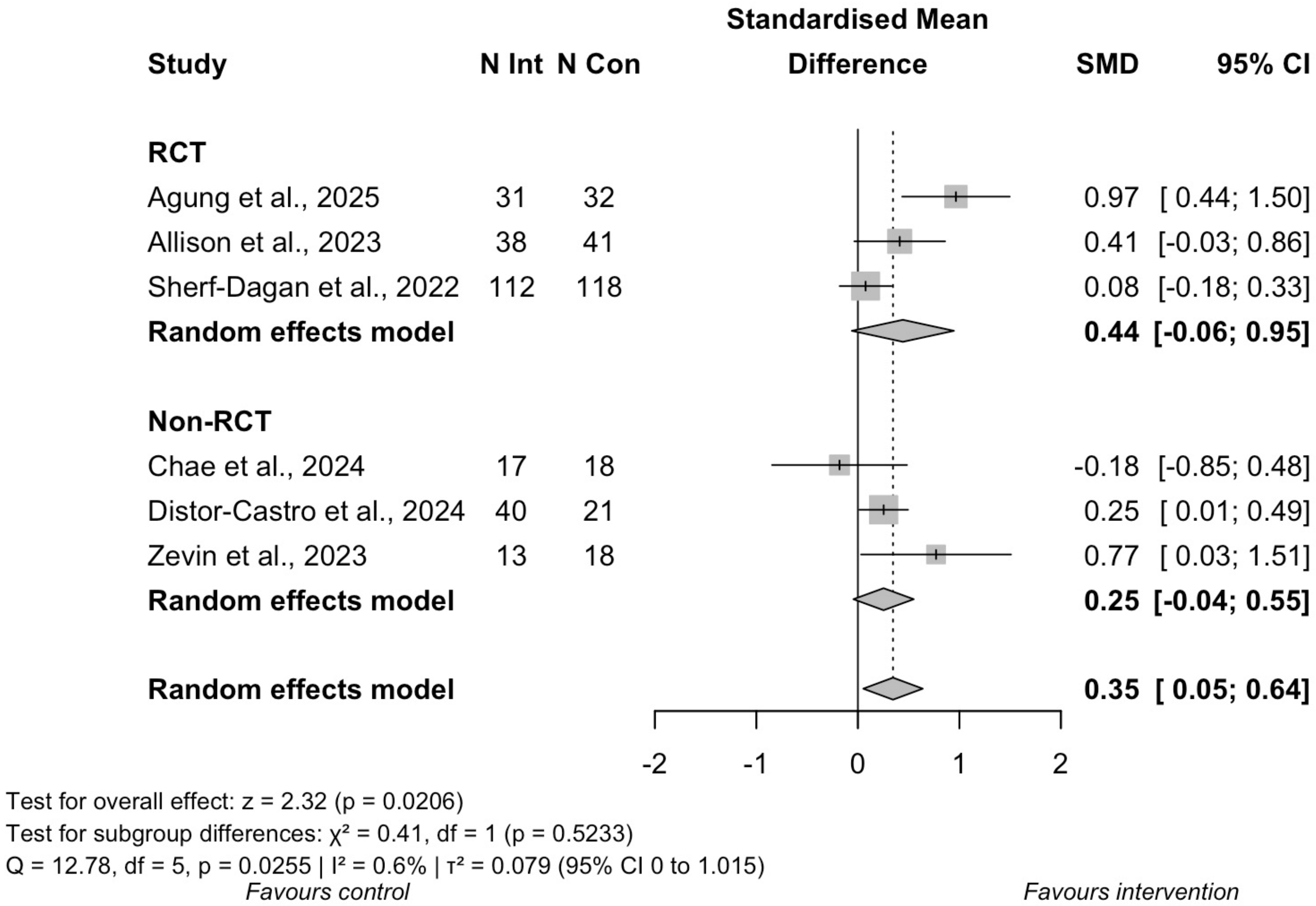
Study
N Int
N Con
Standardised Mean Difference
SMD
95% CI
RCT
Agung et al., 2025 31 32 0.97 [0.44; 1.50]
Allison et al., 2023 38 41 0.41 [-0.03; 0.86]
Sherf-Dagan et al., 2022 112 118 0.08 [-0.18; 0.33]
Random effects model 0.44 [-0.06; 0.95]
Non-RCT
Chae et al., 2024 17 18 -0.18 [-0.85; 0.48]
Distor-Castro et al., 2024 40 21 0.25 [0.01; 0.49]
Zevin et al., 2023 13 18 0.77 [0.03; 1.51]
Random effects model 0.25 [-0.04; 0.55]
Random effects model 0.35 [0.05; 0.64]
-2
-1
0
1
2
Test for overall effect: z = 2.32 (p = 0.0206)
Test for subgroup differences: χ² = 0.41, df = 1 (p = 0.5233)
Q = 12.78, df = 5, p = 0.0255 | I² = 0.6% | τ² = 0.079 (95% CI 0 to 1.015)
Favours control
Favours intervention

(d)

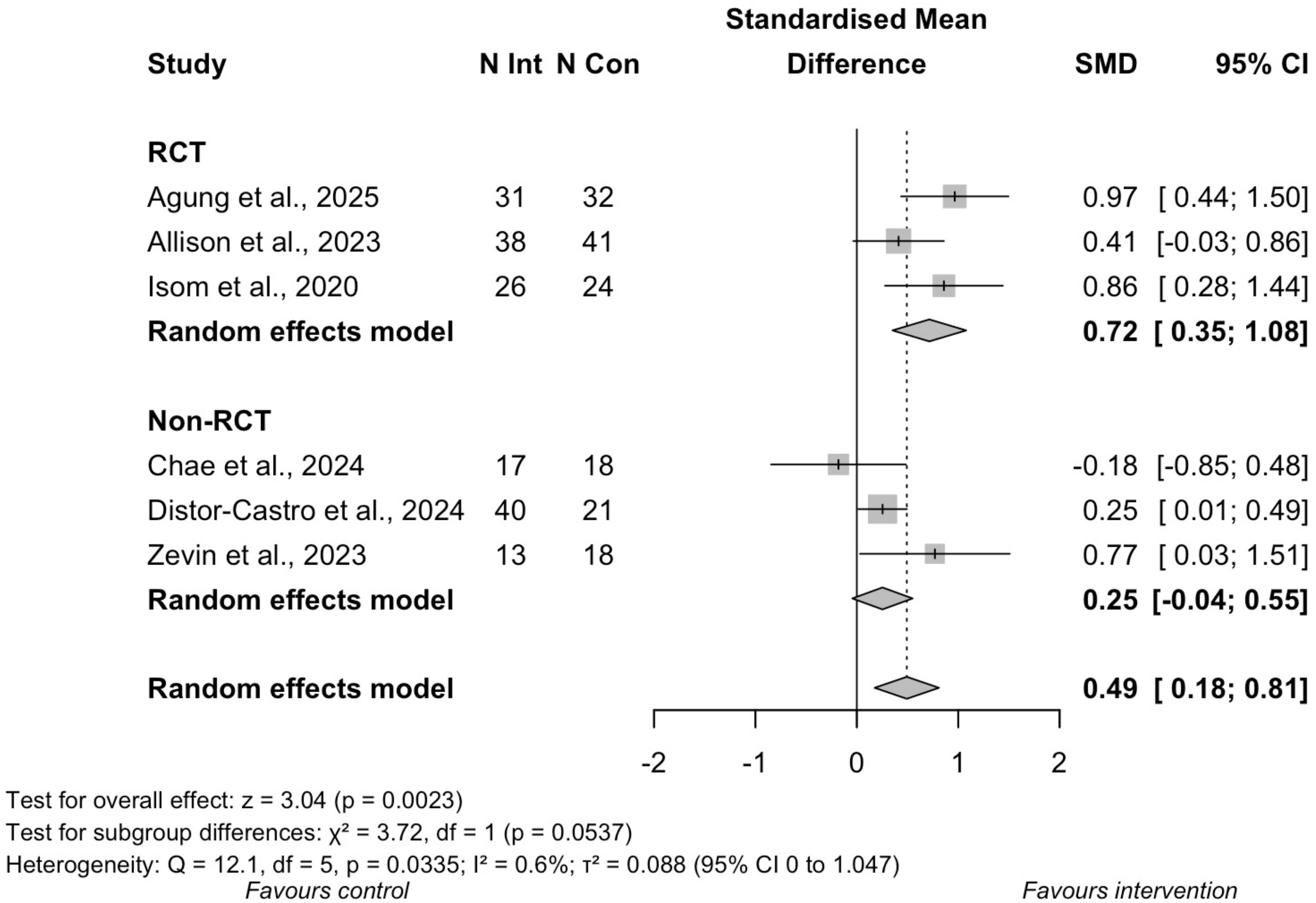
Standardised Mean
Study
N Int
N Con
Difference
SMD
95% CI
RCT
Agung et al., 2025 31 32 0.97 [0.44; 1.50]
Allison et al., 2023 38 41 0.41 [-0.03; 0.86]
Isom et al., 2020 26 24 0.86 [0.28; 1.44]
Random effects model 0.72 [0.35; 1.08]
Non-RCT
Chae et al., 2024 17 18 -0.18 [-0.85; 0.48]
Distor-Castro et al., 2024 40 21 0.25 [0.01; 0.49]
Zevin et al., 2023 13 18 0.77 [0.03; 1.51]
Random effects model 0.25 [-0.04; 0.55]
Random effects model 0.49 [0.18; 0.81]
-2
-1
0
1
2
Test for overall effect: z = 3.04 (p = 0.0023)
Test for subgroup differences: χ² = 3.72, df = 1 (p = 0.0537)
Heterogeneity: Q = 12.1, df = 5, p = 0.0335; I² = 0.6%; τ² = 0.088 (95% CI 0 to 1.047)
Favours control
Favours intervention

(e)

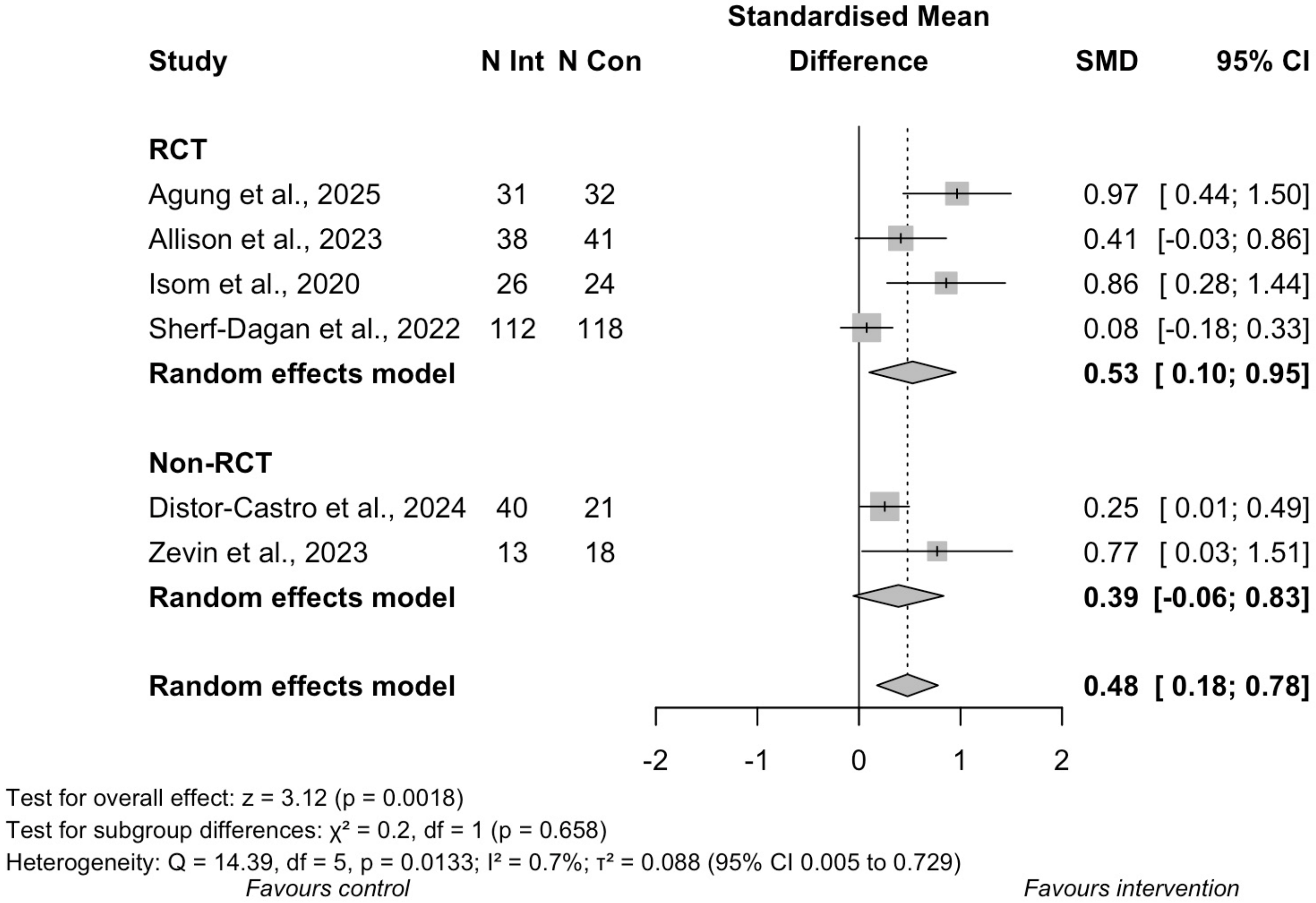
Study
N Int
N Con
Standardised Mean Difference
SMD
95% CI
RCT
Agung et al., 2025 31 32 0.97 [0.44; 1.50]
Allison et al., 2023 38 41 0.41 [-0.03; 0.86]
Isom et al., 2020 26 24 0.86 [0.28; 1.44]
Sherf-Dagan et al., 2022 112 118 0.08 [-0.18; 0.33]
Random effects model 0.53 [0.10; 0.95]
Non-RCT
Distor-Castro et al., 2024 40 21 0.25 [0.01; 0.49]
Zevin et al., 2023 13 18 0.77 [0.03; 1.51]
Random effects model 0.39 [-0.06; 0.83]
Random effects model 0.48 [0.18; 0.78]
-2
-1
0
1
2
Test for overall effect: z = 3.12 (p = 0.0018)
Test for subgroup differences: χ² = 0.2, df = 1 (p = 0.658)
Heterogeneity: Q = 14.39, df = 5, p = 0.0133; I² = 0.7%; τ² = 0.088 (95% CI 0.005 to 0.729)
Favours control
Favours intervention

(f)

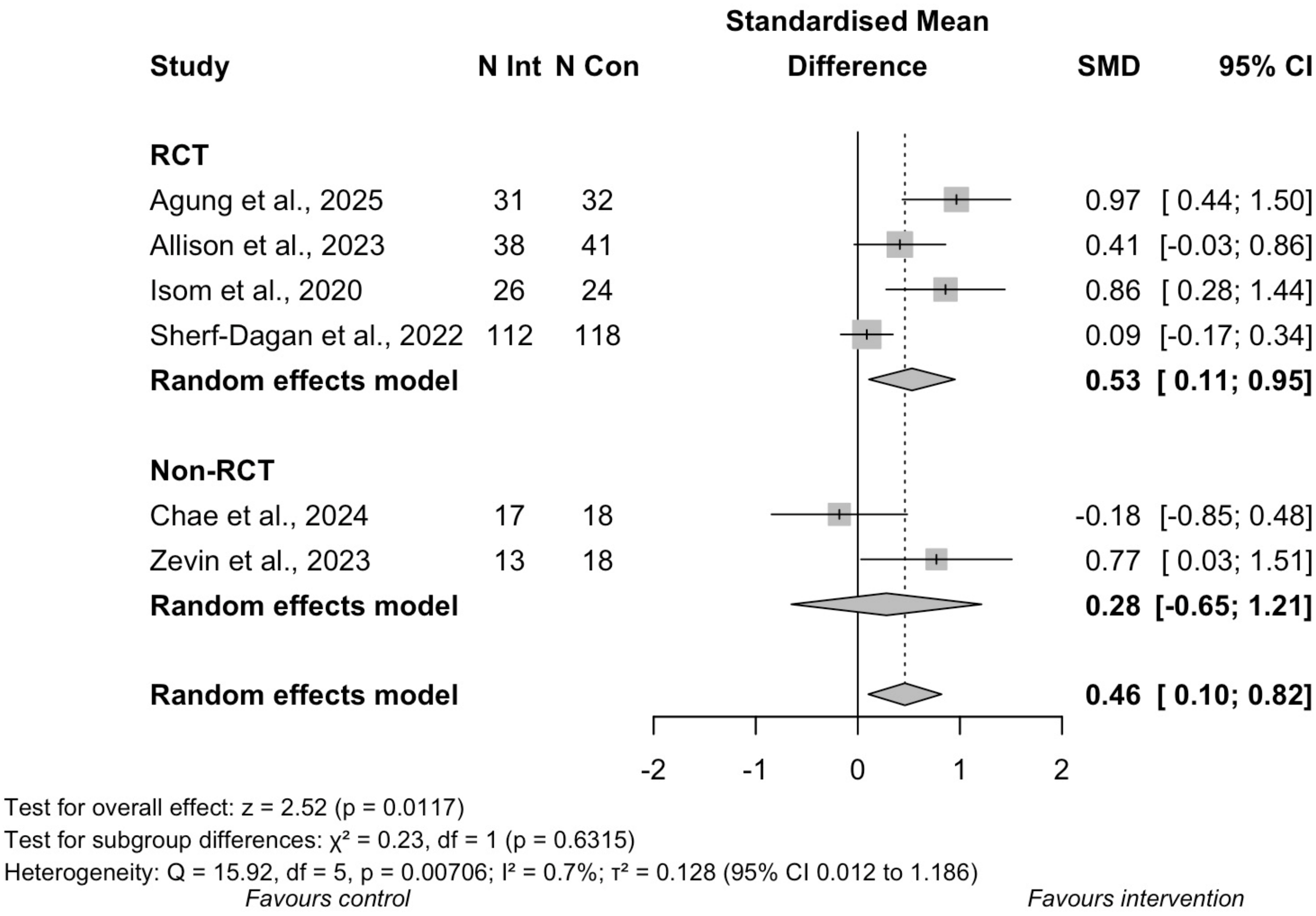
Standardised Mean
Study
N Int
N Con
Difference
SMD
95% CI
RCT
Agung et al., 2025 31 32 0.97 [0.44; 1.50]
Allison et al., 2023 38 41 0.41 [-0.03; 0.86]
Isom et al., 2020 26 24 0.86 [0.28; 1.44]
Sherf-Dagan et al., 2022 112 118 0.09 [-0.17; 0.34]
Random effects model 0.53 [0.11; 0.95]
Non-RCT
Chae et al., 2024 17 18 -0.18 [-0.85; 0.48]
Zevin et al., 2023 13 18 0.77 [0.03; 1.51]
Random effects model 0.28 [-0.65; 1.21]
Random effects model 0.46 [0.10; 0.82]
-2
-1
0
1
2
Test for overall effect: z = 2.52 (p = 0.0117)
Test for subgroup differences: χ² = 0.23, df = 1 (p = 0.6315)
Heterogeneity: Q = 15.92, df = 5, p = 0.00706; I² = 0.7%; τ² = 0.128 (95% CI 0.012 to 1.186)
Favours control
Favours intervention

(g)

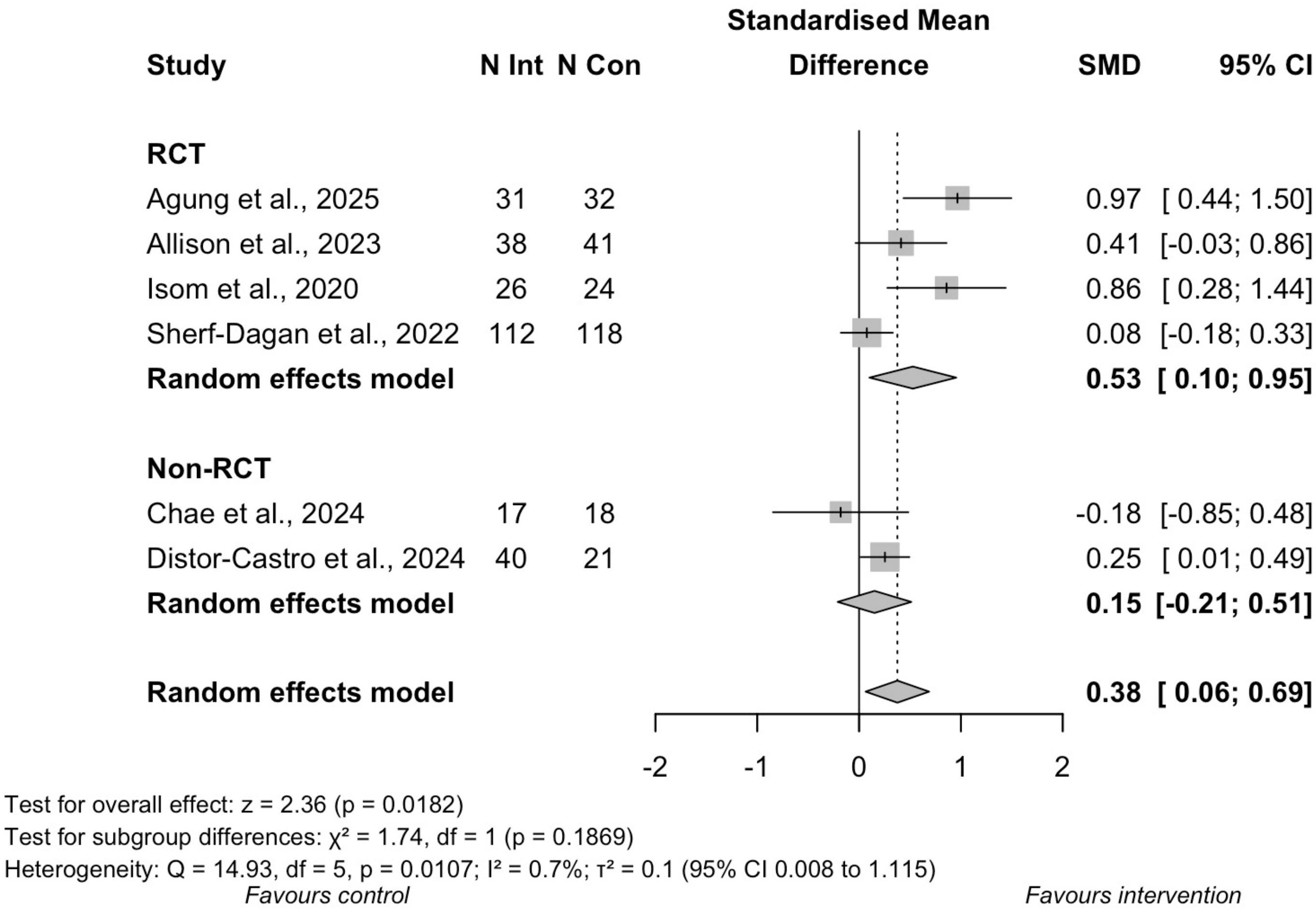

Figure 1. Forest plots from sensitivity analysis, showing pooled effect sizes with each study omitted in turn

Forest plots show the effect of omitting each study in turn: (a) omitting Agung et al. (b) omitting Allison et al., (c) omitting Isom et al., (d) omitting Sherf-Dagan et al., (e) omitting Chae et al., (f) omitting Distor-Castro et al., (g) omitting Zevin et al. Results indicate that the overall pooled effect size remained consistent across all iterations.
